# The evaluation of operating Animal Bite Treatment Centers in the Philippines from a health provider perspective

**DOI:** 10.1371/journal.pone.0199186

**Published:** 2018-07-12

**Authors:** Anna Charinna B. Amparo, Sarah I. Jayme, Maria Concepcion R. Roces, Maria Consorcia L. Quizon, Ernesto E. S. Villalon, Beatriz P. Quiambao, Mario S. Baquilod, Leda M. Hernandez, Louise H. Taylor, Louis H. Nel

**Affiliations:** 1 Global Alliance for Rabies Control, Sta. Rosa City, Philippines; 2 South Asia Field Epidemiology and Technology Network (SAFETYNET), Quezon City, Philippines; 3 Disease Prevention and Control Bureau, Department of Health, Manila, Philippines; 4 Research Institute for Tropical Medicine, Department of Health, Muntinlupa City, Philippines; 5 Global Alliance for Rabies Control, Manhattan, New York, United States of America; 6 University of Pretoria, Pretoria, South Africa; Wistar Institute, UNITED STATES

## Abstract

**Background:**

The Philippine government has an extensive network of 513 Animal Bite Treatment Centers (ABTCs) to supply rabies post exposure prophylaxis (PEP), reaching over 1 million bite victims in 2016. The network was evaluated using a review of existing national and provincial data, key informant interviews and surveys in sample ABTCs to determine the cost-effectiveness of this network in preventing human rabies deaths.

**Methodology and principal findings:**

One urban and one rural ABTC in each of three selected provinces were studied in more detail. PEP delivery generally followed national guidance based on best practices, but there was evidence of operational challenges in supplying all ABTCs with adequate biologics and recently trained staff. Funding was contributed by different levels of government and in some clinics, patients paid for a significant fraction of the total cost. From a health provider perspective including both fixed and variable costs, the average PEP course delivered cost USD 32.91 /patient across urban ABTCs (with higher patient throughput) and USD 57.21 /patient across rural ABTCs. These costs suggests that PEP provision in the Philippines cost USD 37.6 million in 2016, with a cost per life saved of USD 8,290. An analysis of the 2,239 suspected rabies deaths from 2008 to 2016 showed no significant decline, and from 2014–16 an average of 8,534 years of life were lost annually. The incidence of rabies deaths from 2014–16 was not clearly related to the provision of ABTCs (per 100,000 population) or human population density, but deaths were more common in higher income provinces.

**Conclusions/Significance:**

In the context of comprehensive rabies control (including dog vaccination and public awareness) ways to reduce this high expenditure on PEP should be explored, to most cost-effectively reach the elimination of human rabies deaths. This paper is accompanied by another containing data on the operation of ABTCs network from a patient perspective.

## Introduction

Wherever rabies is endemic in dogs, potentially exposed bite victims need to be able to access PEP quickly and without great expense. Few rabies endemic countries have been as successful in decentralising the provision of PEP as the Philippines. Here an extensive network of Animal Bite Treatment Centers and trained staff was developed and vaccine is typically provided free of charge to patients.

As the international community seeks to eliminate human deaths from dog-mediated rabies by 2030, there is a need to document existing rabies control programmes so that information about their implementation and cost-effectiveness can be gathered, lessons learned and efforts replicated more widely. Gavi, the Vaccine Alliance, is also currently considering adding rabies vaccine to the portfolio of vaccines that it provides to the low income countries (located in Africa, Asia and Latin America) that it supports.

The Philippines indicated its commitment to rabies control measures by passing the Anti-Rabies Act of 2007 (Republic Act No. 9482) which specified the development of a National Rabies Prevention and Control Program (NRPCP). Components of the national program include: mass vaccination of dogs; dog population management; health promotion; advocacy on responsible pet ownership; establishment of central database system for dogs; provision of pre-exposure prophylaxis (PrEP) for high-risk individuals and post-exposure prophylaxis (PEP) after an exposure from potentially rabid animals [1]. The Department of Health (DOH) was mandated to ensure the availability and adequate supply of WHO pre-qualified human anti-rabies vaccines (ARV) in Animal Bite Treatment Centers (ABTC) at all times.

The Philippines was one of the first countries to shift to the cost saving intradermal administration of PEP in 1997 [1]. A decentralised system of ABTCs (health facilities owned by the national or local government units providing PEP to animal bite patients with possible rabies exposures in accordance with the DOH recommended management protocol) has been expanded greatly over recent years to allow easier access to treatment for bite victims and consequently minimise the public health impact of rabies in dogs. The DOH, through the NRPCP, provides only high quality, imported ARV and equine Rabies Immunoglobulin (eRIG) for the facilities. Human RIG is not provided.

The government-run ABTC network operates alongside Animal Bite Centers (ABCs) which are operated and owned by private individuals or companies/corporations and are not provided with ARV and eRIG by the NRPCP. Whilst ABTCs use intradermal administration, ABCs usually use intramuscular administration for ARV.

This evaluation was designed to describe the roll out of decentralised PEP provision by the government of the Philippines and to assess its impact on animal bite treatment and on human rabies deaths. It aimed to describe how the system currently operates, to assess the costs and the health impacts achieved for the benefit of the Philippines national rabies committee, WHO and Gavi and other countries considering improving rabies control efforts. It was designed to collate information from the national down to the community level to present a full picture of the benefits and challenges of the system. It also sought to describe in more detail the operation of ABTCs in urban and rural settings in three distinct geographical settings (one mountainous province, one lowland province, one island province) likely applicable across many rabies endemic countries.

## Methods

This analysis focused exclusively on the provision of PEP through government run ABTCs, as government provided services are the most likely model to be replicated in low income rabies endemic countries, and to be potentially supported by Gavi. Private services, especially those using intramuscular administration are unlikely to be affordable to the majority of communities living in low income countries where the bulk of human rabies deaths occur. In addition, there is no centralised data on privately–run ABCs in the Philippines.

### Review of key documents and data and key informant interviews

A full list of the documents and data sets reviewed is provided in the supplementary data (Table A in S1 Table). Briefly the reviewed materials included (1) the Anti-Rabies Act of 2007 (2) DOH and Department of Agriculture (DA) administrative orders (3) guidelines concerning the control of rabies, the operation of ABTCs and the provision of PEP (4) relevant datasets on canine rabies cases (5) ABTCs and animal bites treated (5) regional bite case numbers (6) national and regional vaccine stocks and facility staff requirements (7) human rabies cases (8) insurance claims for rabies PEP (9) provincial dog vaccination numbers (9) provincial ABTC staff records (10) vaccine and bite victims treated, as collated from records held at relevant national, regional and provincial Departments of Health and Agriculture.

Population data and income classifications for provinces and municipalities were taken from the Philippine Standard Geographic Code based on the 2015 Census of Population [2]. Life expectancy data for the Philippines was taken from the World Bank Indicators [3]. Daily minimum wage rates data was based on the 2017 list of the Department of Labor and Employment [4].

To gather insights regarding the operations of and access to ABTCs a total of 41 key personnel were interviewed from the national government, Region II, III and IV-B DOH regional offices, the Provincial Health Offices of Nueva Vizcaya, Tarlac and Palawan, 6 ABTC facilities and 3 barangay (village) offices. The interviews were conducted between February and July 2017 (Table B in S1 Table).

### Choice of three provinces for further study

Three provinces were selected based on a good track record of data submission, and reflecting a range of different human population densities and geographies most applicable to Gavi-eligible countries in Africa and Asia (Table 1).

**Table 1:** Description of study provinces.

Province	Administrative divisions	Geography	Population in 2015 from census records	Area (km2)	Population density in 2015 (/km2)
Nueva Vizcaya	275 barangays in 15 municipalities, no highly urbanized cities	Mountainous, mostly rural	452,287	3,975.67	110
Palawan	433 barangays in 23 municipalities and 1 highly urbanized city	Island, mostly rural	1,104,585	17,030.75	65
Tarlac	511 barangays in 17 municipalities and 1 highly urbanized city	75% Lowland, urban and rural	1,366,027	3,053.60	450

### Study ABTCs

To have a better representation of different conditions and patient throughput rates of ABTCs across the chosen provinces, one ABTC situated in the province capital and one in a rural municipality were included (Table 2).

**Table 2:** Description of study ABTCs.

Province	ABTC	Classification	Location	Established	Average number of patients treated per month
Nueva Vizcaya	Nueva Vizcaya Provincial Health Office	Urban	Bayombong Municipality, (Provincial Capital)	2005	200–360 (2012–15), 590 (2016)
	Alfonso Castañeda Rural Health Unit	Rural	Alfonso Castañeda Municipality, 5 hours travel by land south of Bayombong	2014	10–13 (2014–15), 13 (2016)
Palawan	Ospital ng Palawan	Urban	Puerto Princesa City	1991	100 (2013–15), 140 (2016)
	Southern Palawan Provincial Hospital	Rural	Brooke’s Point Municipality, 4 hours travel by land south of PPC	2010	30–70 (2012–15), 80 (2016)
Tarlac	Tarlac Provincial Health Office	Urban	Tarlac City	1994	400–680 (2012–15), 780 (2016)
	Paniqui General Hospital	Rural	Paniqui Municipality, 30 minutes travel by land from Tarlac City	2016	12 (2016)

The study ABTCS have been providing all doses of vaccine to patients for free since the start of 2016 when this became government policy [5]. Prior to the policy change, two of the four doses were provided free, and patients were also charged for vaccine if government supplies vaccine supplies ran out. Since 2015, the Alfonso Castañeda ABTC has had no in-house medical doctor, and was therefore unable to administer RIG to bite patients. Patients requiring RIG have been referred to the Provincial Health Office or other ABTCs in the neighboring province of Nueva Ecija. Ospital ng Palawan in Palawan is one of the few hospitals retained by the national Department of Health and is run independently from the local government; the rest of the study ABTCs were run by local governments. The Alfonso Castañeda and Paniqui ABTCs also receive patients from the nearby province of Nueva Ecija, and Paniqui stores and administers vaccine paid for by the Nueva Ecija local government. The Paniqui ABTC was the most recently opened ABTC studied and due to the small number of patients seen daily, ARVs are sometimes administered intramuscularly (IM) instead of the usual ID route.

### Cost analysis

To gain a clearer understanding of the costs involved in ABTC operations, we collected data on costs for each of the six ABTCs included in the study.

All costs associated with PEP during 2016 at all levels (national, regional and local) levels were collated (including costs for patients’ initial and all follow-up visits). Variable cost categories included Vaccines (ARV and eRIG) and other consumables (syringes, tetanus toxoid and anti-tetanus serum, antibiotics, logbooks). Fixed costs included personnel (salaries and other personnel costs including training of ABTC staff), vaccine storage (cold chain maintenance and equipment) and information, education, and communication campaign costs. The funding sources for each cost were also recorded to be able to calculate the relative contribution of different levels of government and patients to the total cost of providing PEP.

In the cost analysis, personnel and staff costs were pro-rated to account for estimated time spent on rabies related work, based on interviews conducted with personnel. Equipment costs were calculated taking into consideration annual costs using simple “straight line” depreciation (procurement cost/working life) and the year when the equipment was procured.

To compute the number of ARV vials, we took the proportion of patients who received purified vero cell rabies vaccine (PVRV) and purified chick embryo cell vaccine (PCECV), as costs vary between the two, and assumed that a patient received the same type of vaccine for all the succeeding doses, if any. The proportions of patients who received vaccine for one, two, three, and four visits were then estimated from reviews of a sample of patient logbooks or from line lists provided by ABTC staff. To compute RIG costs, it was assumed that two vials were used for patients above 15 years old and 1 vial for patients 15 years old and younger.

Funding sources included the DOH, local governments, donors, Philippine Health Insurance Corporation (PhilHealth) reimbursements and patients’ out-of-pocket expenses (OOPE).

Number of lives saved was calculated by assuming that 2.2% of patients were bitten by a rabid dog, as per recent estimates by the Research Institute for Tropical Medicine [6] and that 19% of people bitten by a rabid dog would die of rabies in the absence of PEP [7]. We assume that the PEP regime was completed by all patients presumed exposed to rabies by a rabid animal (“at risk” patients), and that there was 100% patient survival after PEP treatment. The total cost of PEP treatment was then divided by the number of lives saved to estimate the cost per life saved.

All costs reported in US dollars (USD) were converted from Philippine Pesos (PHP) based on the exchange rate PHP 47.12: USD 1 as of June 30, 2016.

### Statistical analysis

Calculation of summary statistics, regression and ANOVA analyses were carried out in Excel 2013.

### Ethics statement

Ethical clearance was granted by the National Ethics Committee of the Philippines Council for Health Research and Development (NEC Code: 2017-008-Taylor-ABTC, Study Title: The Evaluation of Operating Animal Bite Treatment Centers in the Philippines).

## Results

### National level operation

A number of legal provisions and operational documents (listed in Table A in S1 Table) describe the prescribed operation of the ABTC network in the Philippines. However, devolution of governance in the Philippines meant that local governments including provinces, cities and municipalities have flexibility in implementing the provision of PEP. They can conduct local communication campaigns, procure additional vaccines for patients and establish additional ABTCs, as long as these meet the DOH requirements.

#### Human resources

At the national level, the NRPCP Manager from the DOH Disease Prevention and Control Bureau (DPCB) has the responsibility for the implementation of the program. Aside from procurement of vaccines and eRIG, DOH also allocates funds to support the shipment of vaccines (to the regional level); personnel trainings; cold storage; information campaigns; database management; monitoring of ABTCs; investigation of Adverse Effects Following Immunization (AEFI); and personnel costs. The Program Manager, together with a team of 4 staff, is also in charge of the allocation and distribution of vaccines to the DOH Regional Offices. The manager is also tasked to monitor utilization of vaccines as well as provide policy base to guide the operations of ABTCs.

At the regional level, a Regional Rabies Medical Coordinator and Regional Rabies Nurse Coordinator are assigned to implement the program. The Regional Rabies Coordinators are tasked to conduct assessment and certification of ABTCs/ABCs; compute vaccine requirement and request to the program; allocate and distribute vaccines to provinces and ABTCs ensuring cold chain; and ensure timely submission of reports.

At the provincial level, the Provincial Rabies Coordinators from the Provincial Health Offices are tasked to compute vaccine requirement and request to the regional office; allocate and distribute vaccines and other logistics of the program; ensure cold chain management of vaccines; and ensure accurate and timely submission of reports to the regional office

At the ABTC level, ABTC personnel are given the responsibility to receive vaccines from the Regional/Provincial Rabies Coordinator; ensure proper cold chain management; screen all bite cases and manage accordingly; maintain an animal bite registry and submit accurate reports to DOH Region and Provincial Health Office on a quarterly basis. The ABTC medical doctor is responsible for monitoring any adverse reactions to the vaccines. At the municipal level, Municipal/City Health Office staff are tasked to screen patients, initiate wound care and refer animal bite cases to ABTCs if necessary.

#### Training needs

Animal Bite Management Training is a requirement for nurses and physicians assigned to the ABTCs. The Research Institute for Tropical Medicine is designated by the DOH as a training center on animal bite management and provides a two and a half day training for ABTC/ABC personnel. The training includes lectures on rabies as a disease in humans and animals; management of animal bites; cold chain management; management of adverse reactions; management of rabies in humans, laboratory diagnosis of rabies and the Rabies Act of 2007. The training also includes hands-on sessions for medical doctors and nurses on intradermal administration of PEP and infiltration of RIG in bite patients. Several DOH Regional Offices also provide similar training.

#### Information, education and communication efforts

At the national level, the Health Promotions and Communication Services (HPCS) of DOH provide assistance in disseminating information regarding the proper health seeking behaviour after a bite and the importance of ABTCs. In 2017, HPCS introduced radio plugs to advertise the importance of consulting ABTCs after an animal bite. DOH Regional Offices also provide and develop information materials such as posters and videos, which are played in the waiting areas of health facilities.

#### Policy support

The Anti-Rabies Act provides the legal basis for the establishment of decentralized ABTCs. DOH DPCB is mandated to release updated guidelines for the management of animal bite and human rabies cases based on the recommendations of WHO, USCDC and other experts through Administrative Orders. The NRPCP Manual of Operations provides detailed guidelines on the establishment and operations of ABTCs [1].

#### Minimum standards for ABTCs

As specified in the Manual of Operations, all ABTCS must: (i) be equipped to provide quality and safe PEP (refrigerator, vaccines, emergency drugs, water supply, waste disposal and record keeping); (ii) provide privacy and comfort for its patients and staff; (iii) be managed by trained doctors and nurses; (iv) follow guidelines to accurately categorize and manage rabies exposures; (v) provide continuous access to safe and effective modern ARV and RIG; (vi) provide PEP to patients by a trained medical doctor and nurse; (vii) post the staff training certificates from a DOH recognized training facility prominently in the health facility; and (viii) maintain an updated rabies exposure registry.

#### Criteria used for the selection of ABTC locations

There is a national target of 1 ABTC/100,000 population and applications for establishment of an ABTC in an area are made by the local government unit and then assessed by the Regional Rabies Coordinator. All animal bite victims are encouraged to visit ABTCs at the soonest possible time after the biting incident, and local government units are encouraged by the DOH to establish ABTCs in underserved areas to make PEP more accessible to patients.

#### Vaccine distribution and demand forecasting

Vaccines are procured annually at the national level from the line budget allotted from the General Appropriations Act of the Department of Budget and Management. Rabies vaccine requirements at the national level and regional level are computed based on the vaccine required to treat the number of Category II and III rabies exposures in the previous year.

Vaccine is centrally stored in the warehouse of the Research Institute for Tropical Medicine, in cold rooms alongside those where EPI vaccines are stored. Vaccine deliveries are verified by the Food and Drug Administration and DOH inspection committees.

Based on the recommendations of the NRPCP Program Manager, vaccines are allocated and distributed every quarter to the DOH Regional Rabies Offices and stored at the regional cold storage (push system). The allocation of vaccines from the regional level to provinces and to ABTC facilities is based upon the previous quarterly utilization and vaccines inventory report submitted. A “no report, no vaccine” allocation rule is implemented by the program at all levels to ensure that reports of the usage of vaccines are completed and submitted to the regional rabies coordinator and from there, the national level. Vaccines are similarly distributed to the provincial/city health offices (and less commonly to some rural ABTCs). Vaccines stored in vaccine refrigerators at provincial health offices are generally further distributed using a pull system where ABTCs collect their allocated vaccine.

Some, but not all, local government units and DOH Regional Offices augment the supply of vaccines coming from the national government, using their own budget allocations. This supplementary vaccine is stored alongside the national DOH supplies, but detailed records of vaccine sources are kept.

#### Recommended vaccine regimens, cost savings and cost sharing

The national guidelines specify that only WHO pre-qualified vaccine should be used for PEP. ARV in the Philippines is delivered almost exclusively using the intradermal route following the modified 2-site (Thai Red Cross) regimen (4 visits on days 0, 3, 7 and 28, 8 doses, 2-2-2-0-2). The small number of patients who have HIV or are immuno-compromised receive 5 doses of vaccines via the Intramuscular route. The differences between the two routes are summarized in Table 3.

**Table 3:** Comparison of recommended intradermal and intramuscular ARV regimens.

	Modified 2-site Intradermal Regimen	Standard Intramuscular Regimen
No. of visits	4 (Days 0, 3, 7, 28)	5 (Days 0, 3, 7, 14, 28)
Amount of vaccine per dose		
PCECV PVRV	0.1 ml 0.1 ml	1 ml 0.5 ml
No. of doses per visit	2	1
Total No. of doses for full regimen	8	5
Total No. of Vials needed per patient for full regimen		
PCECV (1 ml/vial) PVRV (0.5 ml/vial)	0.8 1.6	5 5
Total vaccine cost* per patient for full regimen		
PCECV PVRV	USD 12.39 USD 12.40	USD 77.45 USD 38.75
Total vaccine cost* per visit		
PCECV PVRV	USD 3.10 USD 3.10	USD 15.49 USD 7.75

*using price of vaccines bought at national level in 2016 which were USD 15.49/vial for PCECV and USD 7.75/vial for PVRV

By using the intradermal (ID) route of administration that uses less vaccine per dose, a single vial of vaccine can be used for multiple patients. Assuming no vaccine wastage and using the vaccine prices paid by the national government, the potential cost savings for using the completed ID regimen were USD 65.06 (PHP 3065.63) per patient using PCECV and USD 26.35 (PHP 1241.61) per patient using PVRV if they complete the recommended full ID regimen. This is equivalent to savings of USD 12.39 (PHP 583.82) per visit using PCECV and USD 4.65 (PHP 219.11) per visit using PVRV. Additional cost savings are realised when patients who are known to have been vaccinated previously are given just two booster doses (1 dose on each of 2 visits). For other patients, if the biting animal is observed to be alive and well 14 days after the biting incident, the PEP can be stopped (generally after 3 visits / 6 vaccine doses).

Prior to 2016, vaccine for 1 to 2 of the 4 visits (usually the 1^st^ and 3^rd^ visit) was provided free to patients, and patients had to pay for RIG (if required) and vaccines for the remaining 2 visits. During 2016, the government started to provide free ARV for all 4 visits. Currently, ABTCS are allowed to charge patients for consumables, such as syringes and for eRIG if required. Patients also need to cover their travel expenses and the cost of their time to attend the ABTC.

#### Reporting from ABTCs to the national monitoring database

With a policy of “no report, no vaccines provided” set by DPCB, all ABTCs must submit timely reports. Reports submitted by ABTCs include the quarterly/annual report of animal bites and cohort analysis report accomplished by the ABTC Nurse which provides data on the details of the animal bite victims (sex, age, geographic location, and category of exposure), anti-rabies vaccine and eRIG used, and status of the biting animal. From the ABTCs, they are submitted and collated by the Provincial and City Rabies Coordinators and then submitted to the Regional Rabies Coordinators and subsequently to the NRPCP.

ABTCs also maintain a Rabies Exposure Registry that contains data on personal information, patient history of exposure, post-exposure prophylaxis, and previously immunized patients or those that had repeated exposures. For bite victims who had previous exposure, completion of the previous PEP regimen will serve as a basis for deciding if the bite victim will only require 2 booster doses or will need to receive the entire course of the PEP regimen.

The National Rabies Information System (NARIS) was developed to facilitate online submission of reports by ABTCs, and increased training to submit data to the updated version (NARIS2) has recently begun. It serves as a database for rabies exposures and rabies cases.

#### Philippines Health Insurance Corporation (PhilHealth) involvement

PhilHealth provides health insurance to current and retired government and private company employees, low income families and individual paying members.

From 2012, PhilHealth has included an Animal Bite Treatment (ABT) package to support the NRPCP by defraying the cost of PEP for animal bite patients who are PhilHealth beneficiaries. The PHP 3,000 (USD 63.67) package covers expenses for outpatient wound care, vaccination supplies, antibiotics, anti-tetanus treatment and the provision of vaccine and RIG to animal bite victims with category III bite wounds, category II wounds to the head and neck or exposures to rabies patients. It does not cover PEP for other categories of wounds, wounds from rodents, guinea pigs and rabbits or pre-exposure prophylaxis.

For ABTCs to be recognized by PhilHealth as Institutional Health Care Providers (IHCP) for the outpatient benefit package of animal bite patients, they must: (i) have a Certificate of Recognition as an ABTC issued by the DOH; (ii) submit a Provider Data Record; (iii) pay the annual fee of PHP 1,000 (USD 21.22) and (iv) provide a performance commitment signed by the head of the facility or Local Chief Executive. Once all the requirements are fulfilled, Phil Health provides a Certificate of Eligibility to Participate to the ABTC.

### The Philippines ABTC network

**Fig 1:**
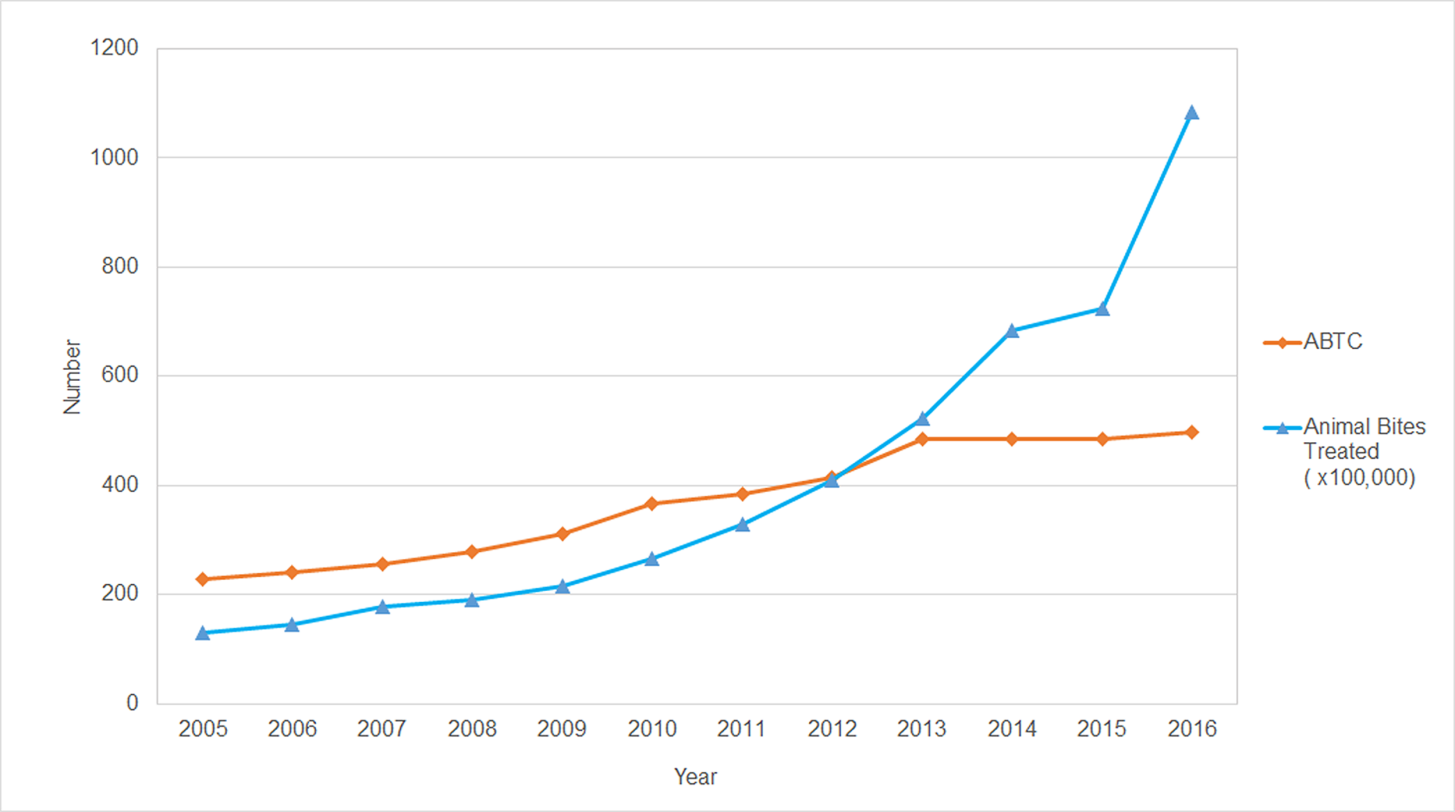
The number of ABTCs and patients treated from 2005–16.

The number of government run ABTCs in the Philippines has been steadily rising since 1997 to a total of 513 by July 2017, and consequently the number of bite patients treated has risen (Fig 1). Records of the numbers of bite patients treated at the many private Animal Bite Clinic facilities in the Philippines are not compiled and consequently were not available for analysis.

For 2016, 770,647 vials (5.242 million ID doses) of anti-rabies vaccines and 89,662 vials of eRIG were procured by the national DOH to supply ABTCs. Compared to the requests received (data on bite cases from 2015), this was expected to meet over 90% of the vaccine demand and 35% of the total RIG needed in 2016.

The government started paying the cost of 2–4 out of 8 doses of vaccine (but not RIG) for patients in 2009. At the start of 2016, the government began paying for all 8 doses of ARV and up to one vial of eRIG per patient, which likely accounted for some of the steep increase in the observed number of patients treated. In 2016, the 1.085 million animal bite patients treated was equivalent to almost 1% of the total population of the country (100.98 million, [2]).

As of July 2017 there were 513 ABTCs in the Philippines. Of the 82 provinces (considering the National Capital Region (NCR) as 1 province), 12 provinces had no ABTCs, 70 provinces had at least one ABTC, with a maximum of 30 (in the NCR).

**Fig 2:**
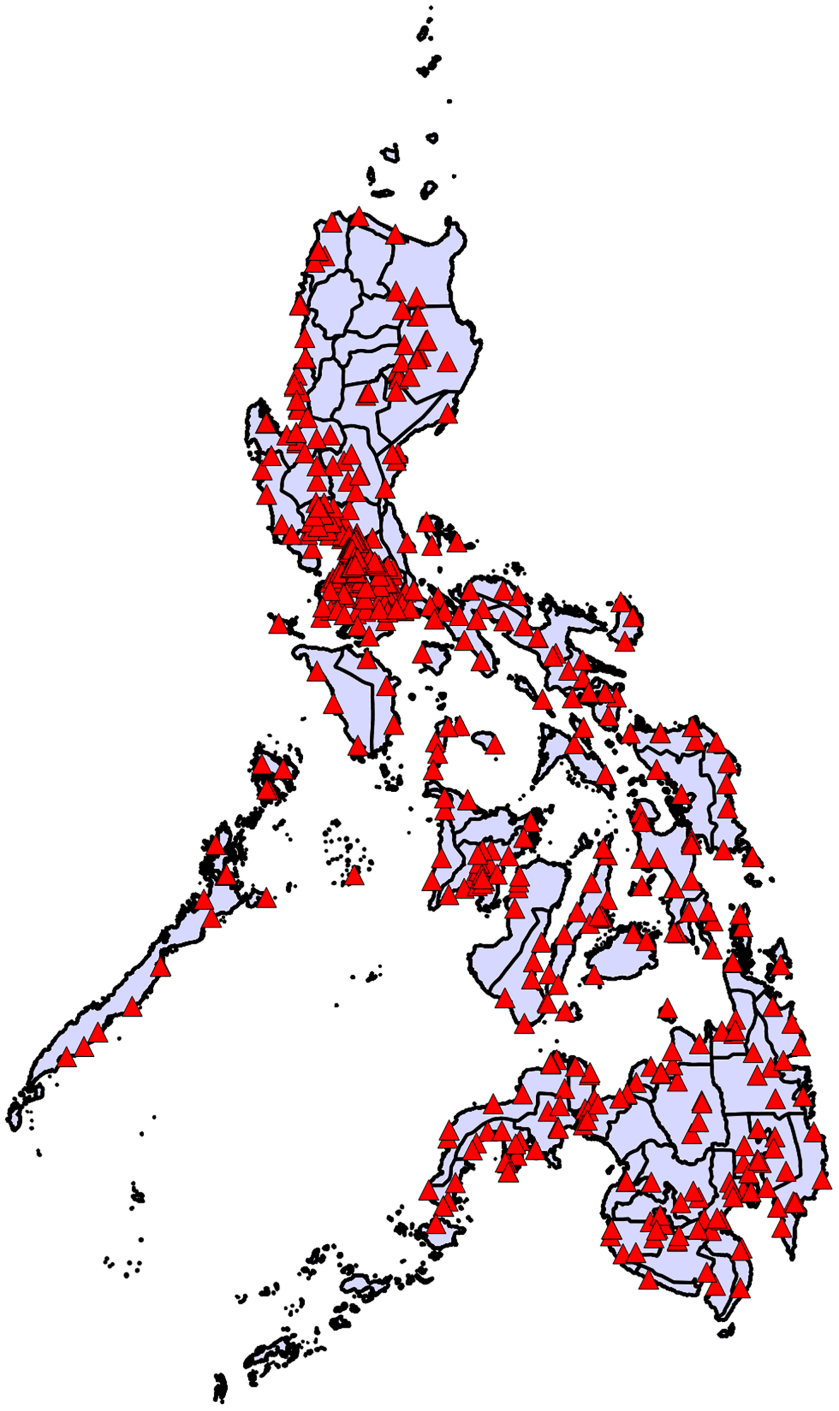
Distribution of ABTCs (per 100,000 population) across provinces.

The number of ABTCs for each province along with the corresponding ABTCs per 100,000 people are provided in S2 Table. Across all provinces, there was a wide variation, with an average of 0.63 and a maximum of 3.15 ABTCs/100,000 population. The distribution of ABTCs /100,000 population is shown in Fig 2. Only 16 provinces have currently reached the target of 1 ABTC per 100,000 population. Reaching the goal of one ABTC/100,000 population for every province will require the establishment of a further 564 ABTCs, more than doubling the current number.

**Fig 3:**
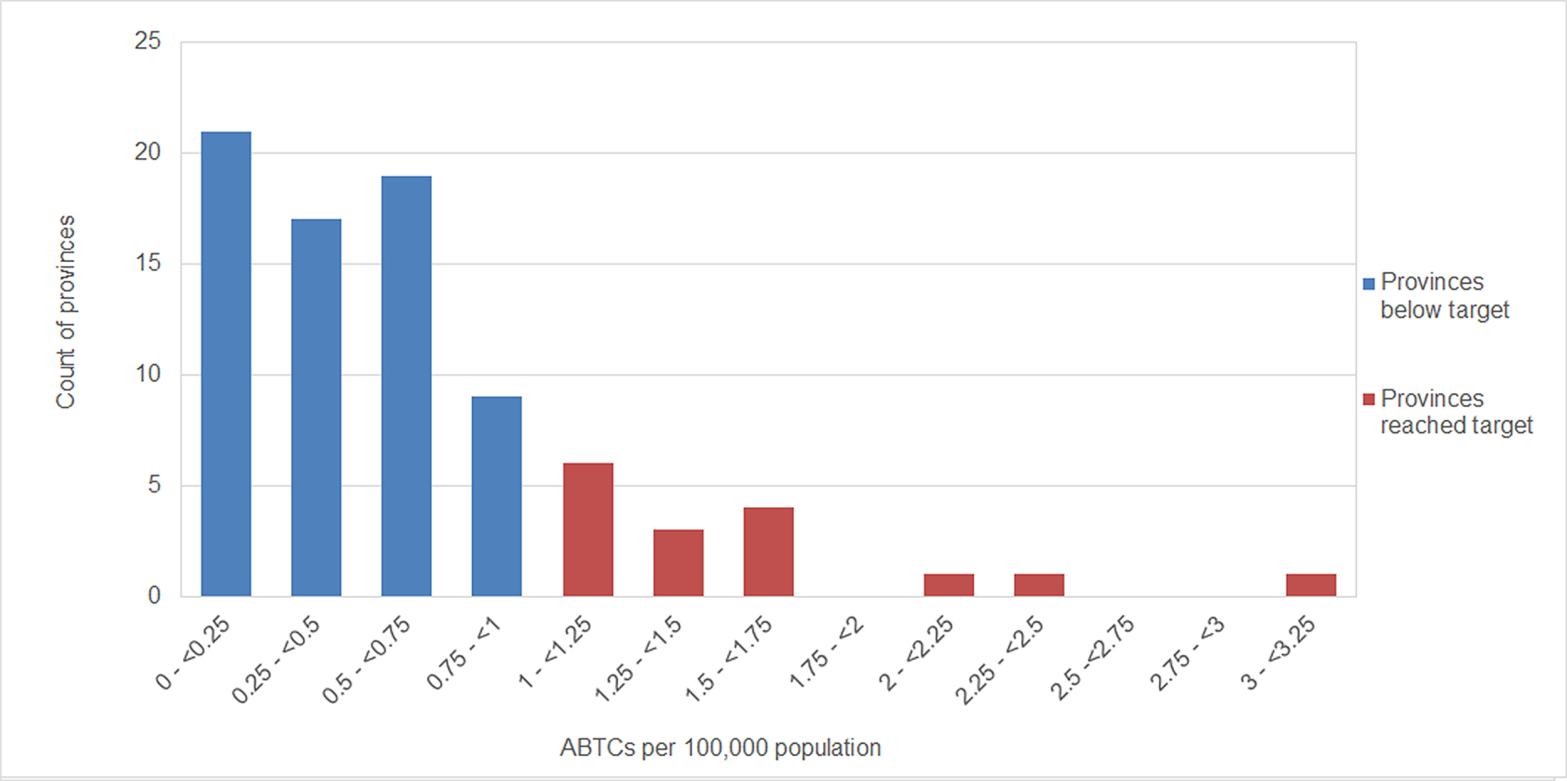
ABTC provision against the income level of the province. NCR = National Capital Region, which has a special income classification.

In the 2015 census, provinces were classified from the 1^st^ income class (richest) to the 5^th^ income class (poorest). The poorest provinces have on average a slightly higher number of ABTCs per 100,000 population, but this trend is not significant (F_4,76_ = 0.374, p>0.5, Fig 3). Thus the distribution of ABTCs is not skewed towards higher income provinces.

Across provinces there was a slight reduction in the number of ABTCs/100,000 population as the human population increased, although given the wide variation this was not significant (correlation coefficient R^2^ = 0.0142). Thus rural and urbanised provinces did not significantly differ in their number of ABTCs / 100,000 people. However, in rural provinces with 100,000 people spread across a larger area, this could still translate into larger distances to the nearest ABTC for patients.

#### Reimbursement of expenses by Phil Health

At the end of June 2016 there were 269 ABTC and ABCs across 74 different provinces accredited by PhilHealth. These provinces had between 1 and 10 accredited ABTCs, except for Palawan (12), Iloilo (17) and Laguna (21).

The total number of claims paid by PhilHealth for PEP is given in Table 4, and has risen from 28,697 in 2014 to 61,219 in 2016. The largest proportion of claims paid was for low-income families. These claims represent 4.2%, 5.9% and 5.6% of the total bites treated in 2014, 2015 and 2016, respectively. A breakdown of the number of patients treated in government run and private clinics was not available, but it is likely that most low income patients would seek treatment in ABTCs.

**Table 4:** Number of claims paid by PhilHealth for eligible bite victim patient costs.

Member Category	Total Number of Claims Paid		
	2014	2015	2016
Government employees	4,959	5,386	6,180
Indigent (low income families)	11,878	19,228	25,960
Individually paying members	5,690	7,554	10,753
Retired members	1,067	4,932	9,051
Overseas Workers Program	690	762	1,040
Private company workers	4,413	5,099	8,235
**TOTAL**	**28,697**	**42,961**	**61,219**

### Provincial level

#### Animal Bite Treatment Centers and rabies cases in the study provinces

A review of records and interviews with provincial rabies program coordinators were carried out to examine the distribution of ABTCs in the three study provinces. The figures used in this section are from lists provided by the provincial offices, which included ABTCs that were established in 2016. Some of these figures do not correspond with the national list provided by the Department of Health (S2 Table), which only includes ABTCs that have been assessed and certified and may not reflect very recently opened ABTCs that are still being assessed for certification.

The number of ABTCs in each of the study provinces and the split between urban and rural municipalities is given in Table 5. Whilst Tarlac has just two ABTCs for over 1.3 million people, Nueva Vizcaya and Palawan have reached the DOH goal of 1 ABTC per 100,000 human population with 5 and 21 operational ABTCs respectively.

**Table 5:** Number of ABTCs and human rabies cases in the 3 study provinces.

Province	Human population	Human density	No. of ABTCs (urban/rural)	Provincial Human Rabies Case data (urban/rural case numbers)					National case data
				2012	2013	2014	2015	2016	2014–16
Nueva Vizcaya	452,287	110/km^2^	5 (4/1)	5 (2/3)	6 (1/5)	3 (1/2)	3 (1/2)	4 (3/1)	5 (2/3)
Palawan	1,104,585	65/km^2^	21 (3/18)	0	0	0	0	0	0
Tarlac	1,366,027	450/km^2^	2 (1/1)	5 (1/4)	6 (1/5)	4 (2/2)	9 (1/8)	6 (0/6)	19 (5/14)

Human rabies cases also varied across the three study provinces with none in recent years in Palawan, but between 3 and 9 per year in the other provinces (Table 5). An increase in rabies cases was noted in 2015 and 2016 in Tarlac, which also has the fewest ABTCs per population. Most cases are from the rural areas (Table 6) where there are fewer ABTCs. Again, the data from the provincial health office shows some minor discrepancies with the national case database, suggesting imperfect reporting to the Department of Health Epidemiology Bureau.

**Table 6:** Reported vaccination coverage of dogs and confirmed animal rabies cases in the 3 study provinces.

Province	Estimated Dog Population*	Animals Vaccinated in 2016	Vaccination Coverage	Animal rabies confirmed by BAI			
				2013	2014	2015	2016
Nueva Vizcaya	50,269	32,080	64%	7	6	3	NA
Palawan	142,283	45,765	32%	7	7	2	8
Tarlac	114,941	47,414	41%	4	6	2	4

Human case numbers were highest in Tarlac (with the least number of ABTCs), and there were no human rabies cases reported in the past 5 years in Palawan, which had the highest number of ABTCs per population (2/100,000) among the 3 study provinces. However, taking account of the population sizes showed that 2014–16 case incidence was similar for Nueva Vizcaya (1.11 / 100,000 people) and Tarlac (1.39 / 100,000 people), even though the former has reached the goal of 1 ABTC / 100,000 population and the latter has not.

**Fig 4:**
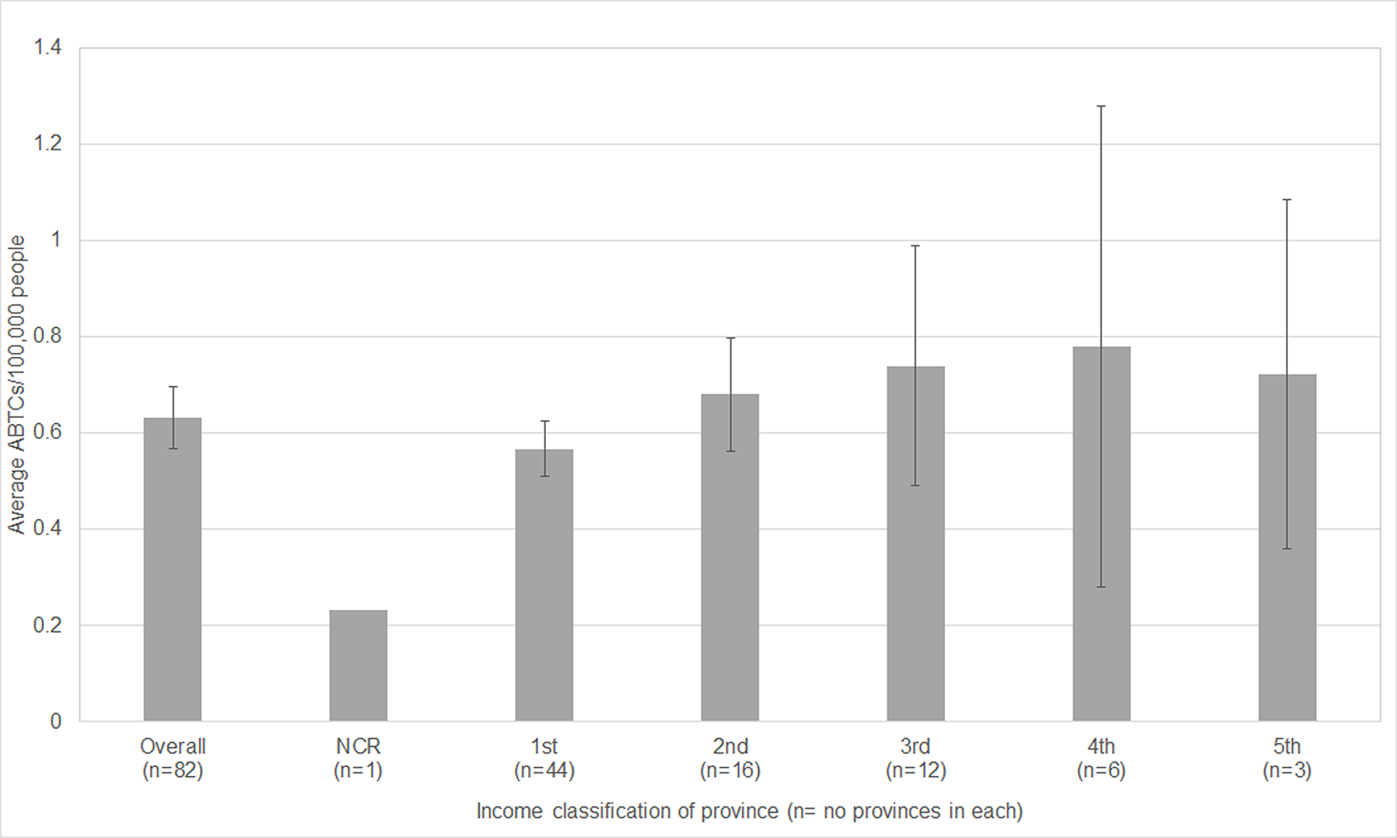
Patients who received PEP in Nueva Vizcaya, Palawan, and Tarlac, 2012–2016. The numbers inset into the bars refer to the number of operational ABTCs.

The number of patients receiving treatment at the ABTCs has been steadily increasing since 2012, which is not very clearly related to the increase in the number of operational ABTCs (Fig 4) and suggests a rising community awareness of the need for PEP. There is a notable increase from 2015 to 2016 in all provinces, which may be partly explained by the provision of free vaccines since the start of 2016.

Listed in Table 6 are the estimated dog vaccination coverages for 2016, and the recent animal rabies cases confirmed by the reference laboratory in the national Bureau of Animal Industry (BAI) for each of the provinces. Animal rabies has been consistently reported since 2012 in the 3 provinces, suggesting a continuing risk of rabies spilling over to humans, even if no human rabies cases are reported.

### ABTC level

#### Animal Bite Treatment Centers included in the study

**Fig 5:**
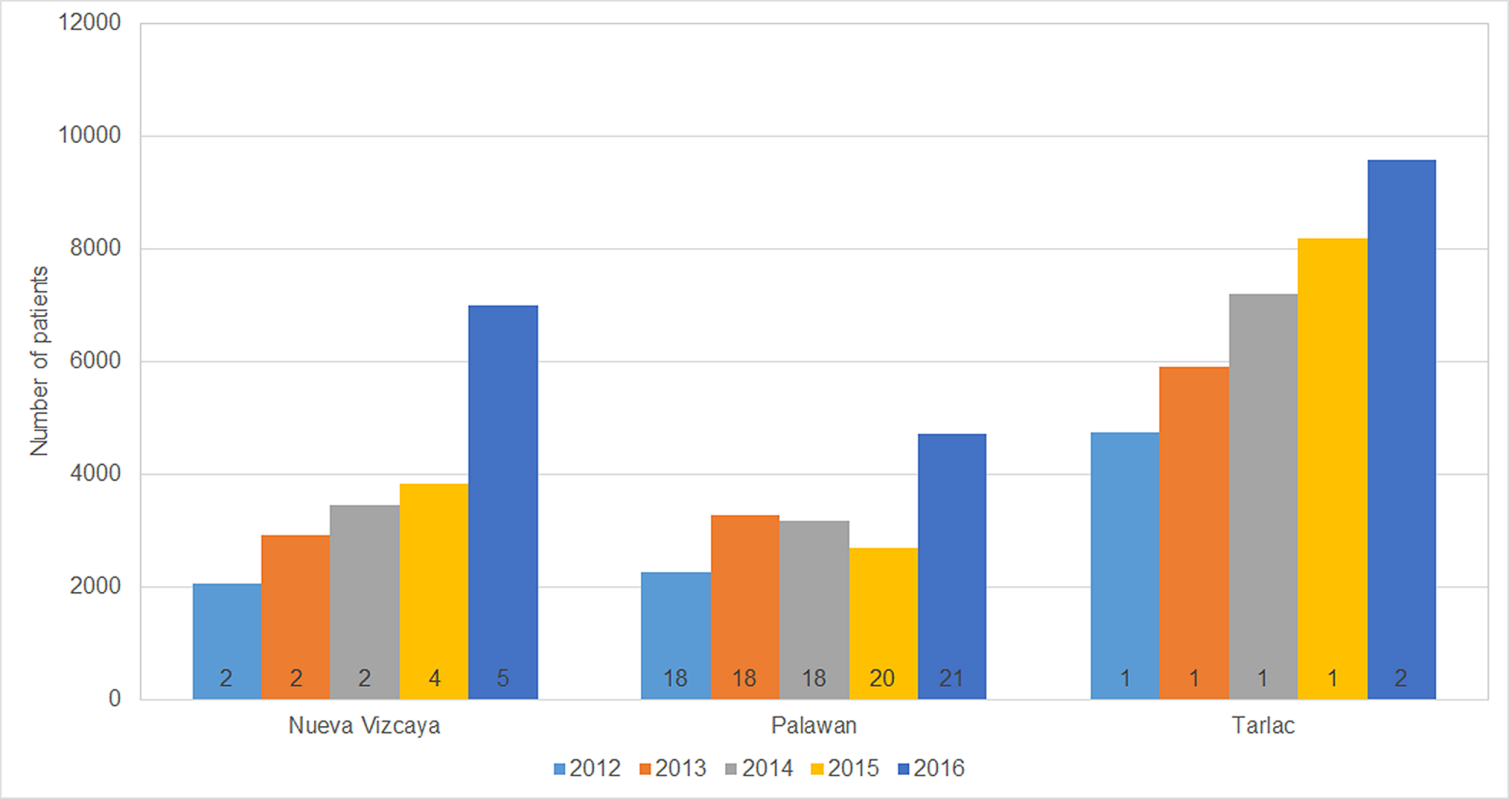
Patients who received PEP in the 6 study ABTCs, 2012–2016.

The number of animal bite patients served in each of the six ABTCs included in the study has been steadily increasing since 2012, suggesting again that awareness of the need for treatment is rising. The most marked increase from 2015 to 2016 occurred at the urban ABTC in Nueva Vizcaya. In all three study provinces, PEP provision was higher at the urban ABTC than the rural ABTC, but this was far less pronounced in Palawan (Fig 5), possibly because there are 2 other ABTCs in Puerto Princesa.

Table 7 shows the cost savings realised per ABTC through the use of the ID regimen for vaccine administration compared with IM administration. This is taking into consideration the actual number of patients served in 2016, the number of visits per patient and the quantity and type of vaccine used (i.e. accurate vaccine wastage is captured). Both of the highest throughput clinics saved over USD 200,000 (PHP 9.424 million) through the use of the ID regimen, and even ABTCs with very small numbers of patients treated yielded savings over USD 2,000 (PHP 94,240). This was partially achieved by asking patients to return on specific days to facilitate vial sharing.

**Table 7:** PCEC and PVRV savings (USD) at the 6 study ABTCs, 2016.

	Nueva Vizcaya		Palawan		Tarlac	
	Urban ABTC	Rural ABTC	Urban ABTC	Rural ABTC	Urban ABTC	Rural ABTC
Number of patients treated	6,132	151	1,260	1,020	9,539	135
PCECV (1ml) vials used	2,845	198	368	472	2,501	18
PVRV (0.5ml) vials used	1,412	61	534	193	5,169	87
Cost savings through ID use of PCECV	212,489	2,184	26,674	35,126	184,011	1,284
Cost savings through ID use of PVRV	20,934	307	7,598	2,847	74,636	1,013
Total Savings in USD	233,422.10	2,491.69	34,271.42	37,972.43	258,646.50	2,297.52

In addition to the savings realized by using an ID regimen to reduce the amount of vaccine and syringes required, patients also save on transportation costs and lost income costs due to the fewer visits required.

#### Vaccine availability and demand

Despite ABTC vaccine requests being based on the previous PEP provision data, if vaccine budgets are low, the vaccine allocated by the national government to ABTCs can be lower than is needed to supply patients. Since health provision is decentralised in the Philippines, for many ABTCs the supply is supplemented by LGU purchases of vaccine. In all of the study ABTCs, government-provided vaccine and eRIG is administered to patients free of charge until it runs out. If no more free product is available, the patients have to purchase it from private pharmacies and bring it to the ABTC for administration.

Data on periods of vaccine stock-outs was not complete for all ABTCs, but reported stock-outs ranged from 2 weeks to 4 months in 2016 at the urban ABTCs in Nueva Vizcaya and Tarlac, and this resulted to stock-outs in the rural counterpart in Nueva Vizcaya as well. Southern Palawan Provincial Hospital (rural ABTC) reported having regular eRIG stock-outs because of the small number of vials allocated to their ABTC.

Table 8 shows the actual number of vials of ARV and eRIG used to treat patients in each of the 6 ABTCs, and the number that had to be paid for by patients themselves. As such information is not recorded, it is not known how many patients never received PEP due to their inability to pay for vaccine. Across the six ABTCs surveyed, respectively 73% (range 37% to 100%) and 30% (range 16% to 100%) of the ARV and eRIG demand was fulfilled by the government (national /regional or provincial).

**Table 8:** Vaccine supplied by the government and paid for by patients, 2016.

	Nueva Vizcaya		Palawan		Tarlac		All
	Urban ABTC	Rural ABTC	Urban ABTC	Rural ABTC	Urban ABTC	Rural ABTC	
**ARV vials**							
Total vials of ARV used	4,257	259	902	665	7,670	144	13,897
Vials of ARV paid for by patients	0	38	41	420	3,135	18	3,652
% of need met by government	100%	85%	95%	37%	59%	88%	73%
Government sources*	N, P	N, P	N, R	N, P, R	N, R, P, CM	N, CM	
**eRIG vials**							
Total vials of eRIG used	1,668	N/A	708	64	2,942	19	5,401
Vials of eRIG paid for by patients	1,408	N/A	538	19	1,814	0	3,779
% of need met by government	16%	N/A	24%	70%	38%	100%	30%
Government sources*	N	N/A	N	N, R	N, R, P, CM	N, R	

*N—national DOH, R—regional DOH, P—provincial government, CM—city or municipal government

With the exception of Paniqui General Hospital, which had a very low eRIG usage, the majority of vials of eRIG used are those paid for by the patient.

Both ABTCs in Palawan reported a 1–2% vaccine wastage. Reasons included having leftover vaccines from vials opened that were used for a smaller number of patients than was expected, and power outages that affected the cold chain. There was no significant wastage reported from the urban ABTC in Nueva Vizcaya because of the large number of patients seen daily. The practice of combining the remaining vaccine from 2 vials to fill 1 syringe also helps eliminate further wastage.

In the rural ABTC in Tarlac, the IM route was used to administer ARVs when the number of patients seen is fewer than the recommended number of patients sharing a vial under the ID route. To avoid wastage of PCEC vaccines, the Provincial Health Office in Nueva Vizcaya provides more PVRV (0.5 mL) vials than PCEC (1.0 mL) to Alfonso Castañeda Rural Health Unit, which sees only an average of 13 patients per month.

#### Challenges related to staffing and training

A shortage of trained staff was observed in some ABTCs. Two ABTCs had only 1 full-time nurse each and at least 1 standby nurse stationed in another hospital section. One of the rural ABTCs did not have a trained medical doctor, and thus did not provide RIG. At this location, patients requiring RIG were advised to travel to the Provincial Health Office (5 hours away by land), or to another ABTC in the nearby province. While there were medical doctors in some facilities, it was usually the ABTC nurse who assessed the patients’ injuries. RIG was administered by the ABTC nurse in one urban ABTC. In some ABTCs, staff reported that their most recent official training was as far back as 2009 and 1997. Local (therefore unofficial) training of staff was being carried out in some centers. This would suggest that any recent changes to the animal bite guidelines from the Department of Health may not be implemented in the ABTCs, although official memoranda regarding updates to guidelines are cascaded down from the national government to the ABTCs.

In the urban ABTC in Tarlac province, the number of patients was so high that waiting times could be up to 4 hours long. This resulted in some patients choosing to not go back for their Day 3 and Day7 doses. Patients also reported having to spend more on meals during ABTC visits.

#### Operational costs for providing PEP

ABTCs are established within existing hospitals or health service facilities, thus there are usually just small capital costs (such as vaccine storage equipment) needed to establish them. Fixed costs (regardless of number of patients served) include personnel, training of ABTC staff in animal bite management, communication campaigns to inform the community of the availability of the service and need for PEP. Variable costs (which depend on the number of patients served) include rabies vaccines, eRIG, and other consumables like syringes, anti-tetanus vaccine, antibiotics, and costs for vaccine distribution.

A full breakdown of the costs and the sources of funding is provided in S3 Table. Overall, variable costs comprised 80% of the total cost for the six ABTCs, and the cost per patient treated was USD 34.65 (PHP 1,633, Table 9). However, there was large variation in this cost across the ABTCs. The cost per patient was much higher in rural areas (average USD 57.21 or PHP 2,696/ patient) where fewer patients were served compared to urban ABTCs (average USD 32.91 (PHP 1,551), but the number of sites was too small to make very firm generalisations. The urban ABTC in Palawan had fewer patients compared to the other urban ABTCs. This is likely because of the presence of other ABTCs located in the same area.

**Table 9:** Summary of operating costs (in USD) by ABTC for 2016.

	Nueva Vizcaya		Palawan		Tarlac		All provinces		
	Urban ABTC	Rural ABTC	Urban ABTC	Rural ABTC	Urban ABTC	Rural ABTC	Urban ABTC	Rural ABTC	Overall
Personnel	21,072	6,149	8,684	9048	36,834	7,325	66,590	22,522	89,112
Training	900	221	1,159	745	115	272	2,174	1,238	3,412
Vaccine Storage costs	1,394	67	134	54	1,649	4,156	3,177	4,277	7,454
ABTC Equipment	4,982	186	854	5120	16,163	8,342	21,999	13,648	35,647
Information, Education, Communication	5	21	4,250	85	1,545	8	5,800	114	5,914
**Fixed Costs in USD (%)**	**28,353 (19%)**	**6,644 (54%)**	**15,080 (26%)**	**15,052 (40%)**	**56,305 (16%)**	**20,103 (82%)**	**99,740 (18%)**	**41,799 (56%)**	**141,539 (22%)**
Rabies Vaccines (ARV & RIG)	109,567	4,925	36,320	17801	283,525	3,691	429,412	26,417	455,829
Vaccine Distribution Costs	4,522	647	1,635	334	3,640	156	9,797	1,137	10,934
Other consumables	4,692	195	3,974	4649	9,609	520	18,275	5,364	23,639
**Variable Costs in USD (%)**	**118,781 (81%)**	**5,767 (46%)**	**41,929 (74%)**	**22,784 (60%)**	**296,774 (84%)**	**4,367 (18%)**	**457,484 (82%)**	**32,918 (44%)**	**490,402 (78%)**
**TOTAL COSTS**	**147,135**	**12,411**	**57,010**	**37,836**	**353,080**	**24,470**	**557,224**	**74,717**	**631,941**
No. of Patients served	6,132	151	1,260	1,020	9,539	135	16,931	1,306	18,237
Cost / patient (USD)	23.99	82.19	45.25	37.09	37.01	181.26	32.91	57.21	34.65

In rural sites in Nueva Vizcaya and Tarlac, that serves fewer patients, the fixed costs such as staff and equipment account for a greater proportion of the expenditure. The fixed cost was higher in the Tarlac rural ABTC (82%) as it only started operations in 2016. Compared to the other older ABTCs, costs for equipment and other capital outlay were higher since these were newly purchased. As this ABTC continues to operate, the fixed costs are expected to approach those of the other rural ABTCs. The costs in the rural ABTC in Nueva Vizcaya are seemingly low for a rural ABTC that treats few patients, since it does not have a medical doctor and does not provide eRIG. Patients who need RIG were referred to the Provincial Health Office or to other ABTCs in the nearby province.

As the number of patients treated increased, variable costs, notably rabies vaccines, accounted for a higher proportion of the operating expenses.

#### Costs or expenditure by source of funds

Funding for staff and vaccine costs was provided in different ways across provinces and even across ABTCS within the same provinces. S3 Table summarizes the detailed operating expenses of the ABTCs by input category and source of funding.

Most of the ABTC operating costs were shouldered by the national and local government agencies. DOH funds were generally used for national and regional staff, training of ABTC staff, procurement, storage and distribution of vaccines and communication campaigns. Local government funds were used for ABTC personnel, transportation costs related to staff attending DOH training workshops, procurement of additional vaccine supplies (particularly in Nueva Vizcaya and Tarlac in this study) and local communication activities. Other contributors are from donor agencies, e.g. UNICEF provided some cold storage equipment.

Some ABTCs (Nueva Vizcaya Provincial Health Office, Ospital ng Palawan, and Southern Palawan Provincial Hospital) receive reimbursements for patients covered by PhilHealth and these are included in the LGU budgets. However, the actual use of these funds (generally used for salaries or consumables) is at the discretion of the local government unit or chief of hospital.

**Fig 6:**
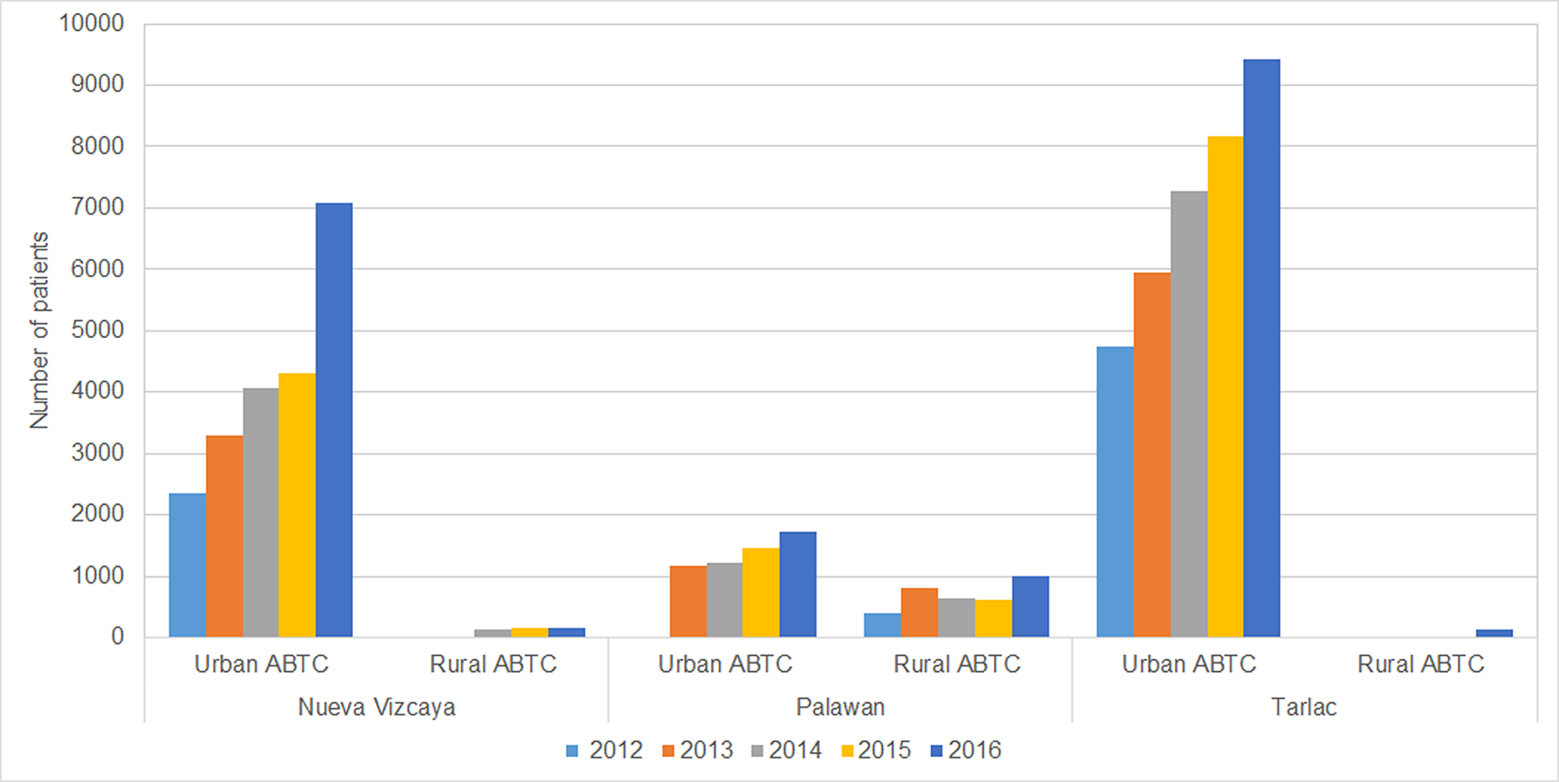
The percentage of ABTC operating costs paid by different funding sources, (A) Nueva Vizcaya, (B) Palawan and (C) Tarlac in 2016.

Fig 6 shows the proportions of funding supplied by source for the different ABTCs. The local government supplied between 12% and 67% of the total operational funds of the ABTCs surveyed (excluding the Palawan urban ABTC run by DOH, S3 Table). Patients themselves usually pay for the syringes needed for vaccine administration, anti-tetanus shots, antibiotics and also vaccines and eRIG when stocks run out at the ABTC. Patients’ out-of-pocket expenses covered a significant fraction (for example 45% and 46% at the urban and rural ABTCs in Palawan respectively, S3 Table) of the total ABTC operational expenses when there are insufficient vaccine and eRIG supplies available. This represents marked inequality in access to free vaccine between and within provinces. OOPE expenses in these graphs are for vaccine or RIG only and do not cover transportation costs and lost income opportunity.

A significant factor affecting the expenditure of different levels of government and patient OOPE, was the cost of vaccine purchased by the different mechanisms involved. Whilst bulk purchasing by the national DOH facilitated a low price per vaccine or eRIG vial, the cost of biologicals to different regional and local government units and patients was up to 4 times higher (Table 10). In part this is due to the high delivery costs to more rural areas.

**Table 10:** Relative costs (in PHP) of vaccine and eRIG through different purchasing mechanisms.

	National DOH	Regional DOH	LGU	Patients (via Pharmacies)
**PCECV 1 ml vial**	730	1,600–1,650	1,600–1,900	1,485–2,800
**PVRV 0.5 ml vial**	365		1,320–1,800	1,320–1,375
**eRIG vial**	920	1,200–1,295	1,500–2,200	1,200–1,700

#### Cost per life saved in the three study provinces

Based on assumptions of 2.2% of patients having been exposed to a rabid animal [6], and that 19% of these would die in the absence of PEP [7], the six ABTCs surveyed were expected to have saved a total of 76 lives in 2016 (Table 11).

**Table 11:** Estimated number and costs (USD) per life saved in the 6 study ABTCs, 2016.

	Nueva Vizcaya		Palawan		Tarlac		OVERALL
	Urban ABTC	Rural ABTC	Urban ABTC	Rural ABTC	Urban ABTC	Rural ABTC	
Total costs of PEP provision in 2016 (USD)	147,135	12,411	57,010	37,836	353,080	24,470	631,941
No. of Patients served	6,132	151	1,260	1,020	9,539	135	18,237
Cost per patient (USD)	23.99	82.19	45.25	37.09	37.01	181.26	34.65
Estimated no. of lives saved	25.63	0.63	5.27	4.26	39.87	0.56	76.23
Cost per life saved (USD) *	5,740.32	19,662.53	10,824.41	8,874.29	8,855.08	43,363.25	8,289.85
Cost per year of life gained^$^	173.42	594.03	327.02	268.10	267.53	1310.07	250.45

*Assuming that 2.2% of patients treated were exposed to a rabid dog, and 19% of these would die without PEP.

^$^ Assuming that on average 1 life lost = 33.1 years of life lost, calculated from section on rabies deaths).

Although there was wide variation across the ABTCs as would be expected for such differences in the patients treated, the overall cost per life saved was 8,289.85 USD (PHP 390,617), and the average cost per year of life gained (equivalent to the cost per DALYs averted) was USD 250.45 (PHP 11,801). Using the per capita GDP for the Philippines of USD 2,951.07 (PHP 139,054.40) in 2016 [8], and the World Health standard of cost-effectiveness (interventions that gain an additional year of healthy life at a cost less than per capita GDP are “very cost-effective” [9,10], provision of PEP in the Philippines at these costs is still a highly cost-effective strategy.

#### Extrapolation across the whole country

The average cost of providing a patient with PEP across the six study ABTCs in 2016 was USD 34.65 (PHP 1632.78, Table 11).

If we assume that this is reflective of the average cost of treating all 1.085 million bite patients across the Philippines, then a simple extrapolation suggests that the total cost of providing PEP across the Philippines in 2016 was USD 37.6 million. (PHP 1.77 billion). This is a very simple generalisation and will not be accurate if the division of patients between urban and rural ABTCs across the whole country is different from that seen here, or if the pattern of different funders purchasing the vaccines varied from that seen here. However, it gives a rough estimation of the cost of the providing PEP across the whole country.

### Failures of the system: Rabies deaths over recent years

#### Human deaths 2008–2016

Despite the substantial increase in the provision of PEP across the Philippines in recent years, the number of deaths due to rabies nationally has only fallen slightly from 306 in 2008 to 260 in 2016 (Fig 1).

**Fig 7:**
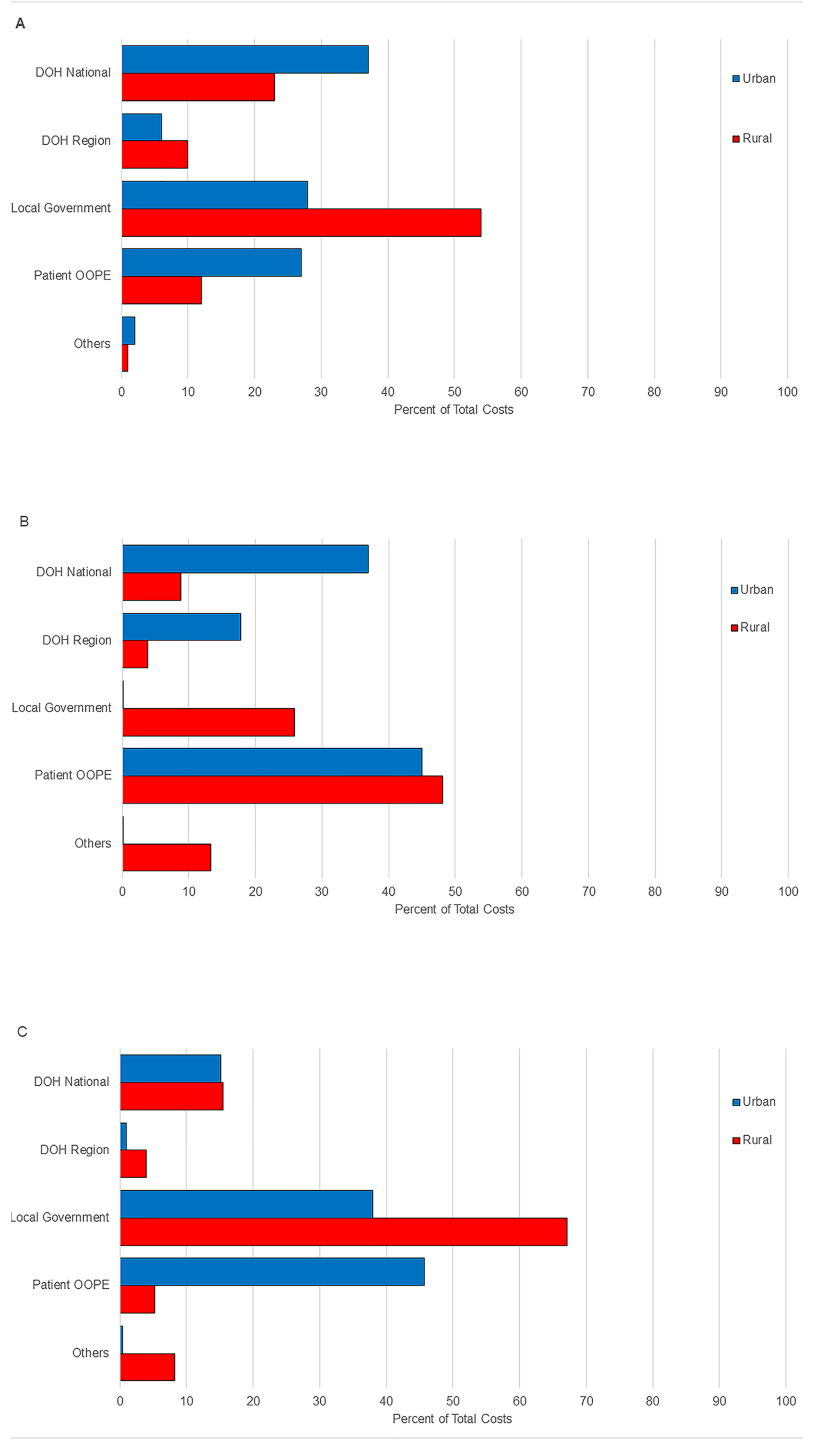
Human rabies cases 2008–16 by sex.

A total of 2,239 human fatalities from rabies have occurred from 2008 to 2016 (Fig 7), corresponding to an average of 248.7 per year, with 72% in males. Whilst the trend line indicates that cases have been declining slightly over the years, the decline is not significant (F_1,8_ = 0.267, p>0.5). Only 2 cases between 2008 and 2016 were laboratory confirmed, and the rest categorised as suspected or probable.

#### Age distribution of human deaths and years of life lost to rabies

**Fig 8:**
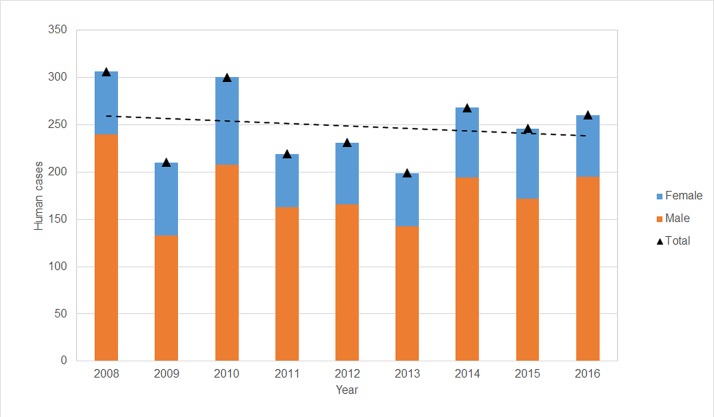
Age distribution of human rabies cases 2008–16.

Age data for the deaths from rabies was available for 2,193 of the cases (Fig 8). 170 of the female deaths (28.0%) and 431 of the male cases (27.2%) occurred below the age of 15. Averaged across 2008–2016, the mean age of deaths was 32.6 for males and 34.3 for females, and the median age of deaths was 33.2 for males and 33.5 for females.

Using average life expectancy (by year and by sex) for the Philippines, the average Years of Life Lost (YLL) to rabies each year between 2008 and 2016 was 8,243, and the average annual YLL from 2014 to 2016 was 8,534, equivalent to 33.1 YLL per case.

#### Pattern of recent deaths

S2 Table gives the number of deaths by province for 2008–2013 and for 2014–16. There was a very weak negative relationship across provinces between the number of ABTCs per 100,000 population and the 2014–16 rabies case incidence / 100,000 population, but this was not significant (R^2^ = 0.0175, F_1,81_ = 1.421, p>0.2, Fig A in S1 Fig). Similarly, there was no relationship across provinces between the human population density and the 2014–16 rabies case incidence / 100,000 population (F_1,80_ = 0.267, p>0.5, Fig B in S1 Fig). In many instances, rabies deaths occurred in municipalities with ABTCs.

**Fig 9:**
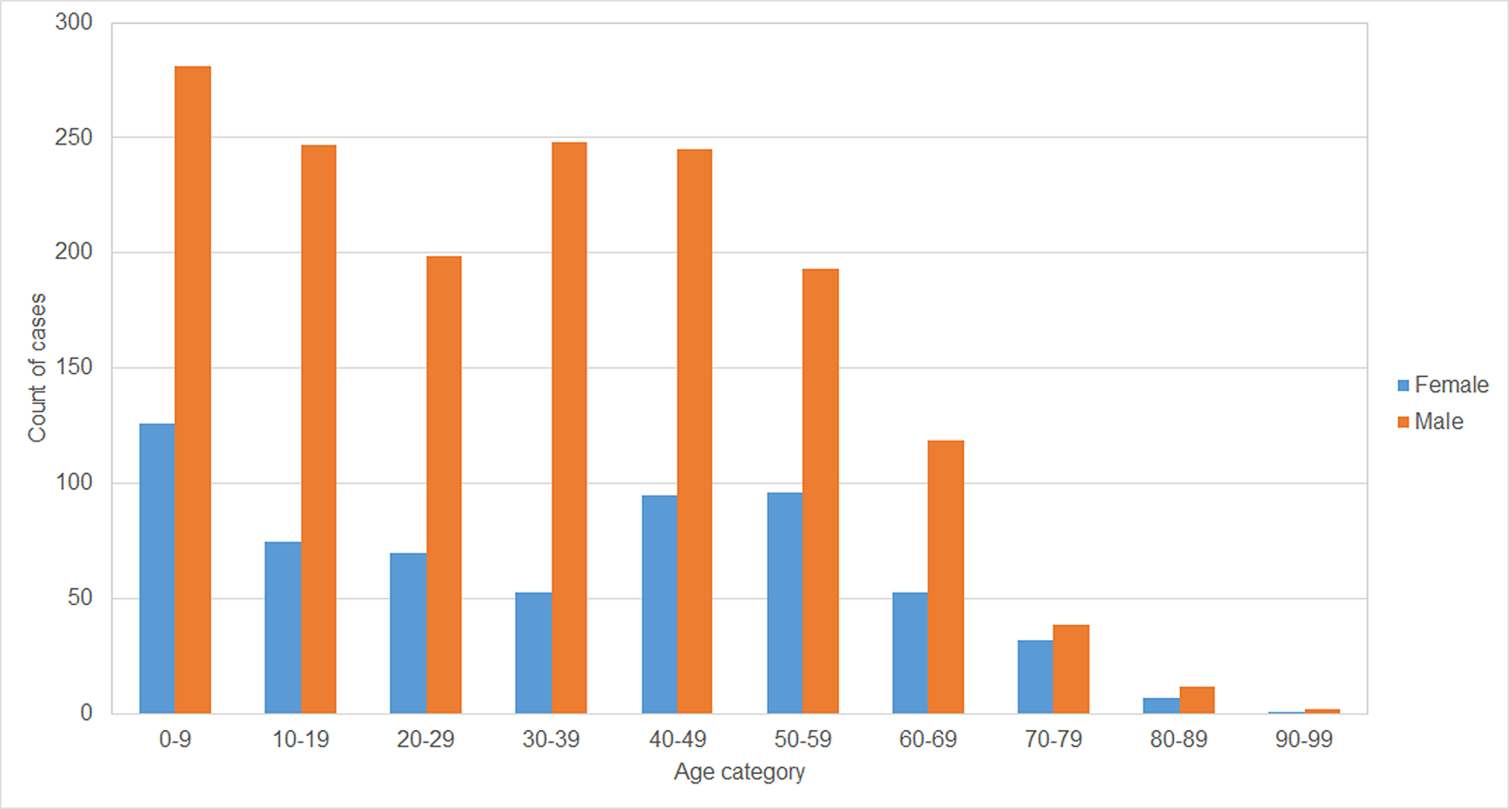
Mean ±SE of recent case incidence for provinces by income class.

The average recent case incidence was calculated for provinces at each income level (Fig 9). If we took into account income classification as per the National Capital Region (NCR), recent case incidence varied significantly between income classes 1 to 5 (F_4,80_ = 4.569, p<0.01). In other words, a person is more likely to die of rabies in provinces with higher income classification.

When analysed at the municipality level, the same trend is apparent, and recent case incidence also declines significantly from high to low income levels (F_5,1610_ = 6.580, p<0.01, S2 Fig).

## Discussion

The Philippines ABTC network has expanded greatly over the last decade, and currently over 500 government-run ABTCs exist (facilitated by cost-sharing mechanism between national and local governments). Private bite treatment centers are also in operation. Although the target of 1 ABTC / 100,000 population has only been reached in 16 provinces, poorer provinces have similar numbers of ABTCs/100,000 people as wealthier ones. Since 2016, these facilities have been providing free anti-rabies vaccines and subsidized eRIG to animal bite/scratch victims.

In 2016, 1.085 million people (more than 1% of the population) received PEP for potential rabies exposures through government ABTCs. A review of the records from six animal bite treatment centers across three provinces revealed large differences in the average cost to provide PEP to a patient, and much variation in the sources of funds utilized. On average it cost USD 34.65 (PHP 1,633) to provide a course of PEP, with costs considerably higher in rural ABTCs treating fewer patients. Government-provided vaccine and particularly eRIG was not available at all times, forcing patients to buy them privately to be administered in the ABTCs. Using recent data on the proportion of patients genuinely exposed to rabies, we estimate that the average cost was USD 8,290 (PHP 390,625) per life saved, or USD 250.45 (PHP 11,801) per year of life gained, still a very cost-effective strategy.

However, human deaths are still occurring in the Philippines, with 260 reported in 2016. At the provincial level, the number of ABTCs / 100,000 population was not significantly associated with a lower incidence of rabies deaths. In addition, the incidence of rabies deaths was higher amongst provinces and municipalities with high-income levels.

### Good guidance, but remaining challenges

The Philippines has good standardised guidance in place to ensure best practice in the provision of PEP, including accreditation of ABTCs, and centralised animal bite management training. ABTCs are well integrated into other health services, being established in existing hospitals and with vaccine distributed through the EPI cold chain facilities. The Philippines is already widely using the cost-saving intradermal regimen and facilitate national level investments in PEP, supplemented by investments from local government level. The policy of “No report, no vaccines” is a good motivator for ABTCs. However, this needs to be partnered with more diligent vaccine usage monitoring /inventory and forecasting (at all levels) to prevent stock-outs.

When PEP demand is rising fast, as has occurred in recent years, vaccine forecasting based on the previous year’s bite data may be inaccurate, and the change of government policy to free provision of all vaccine doses likely caused demand to rise above expectations. Vaccine forecasts are not completely supplied by national government and weeks of unavailable DOH vaccines require emergency procurements of the local government or vaccine purchases by patients, neither of whom are able to access the same competitively priced vaccine as the national government, which further reduces availability. Even across the small number of ABTCs surveyed, vaccine stock-outs were relatively common with 73% of vaccine demand and just 20% of RIG demand being met by the government. It is possible that these stock-out were more prominent in 2016, when a policy change (increasing the number of doses provided free to patients) more dramatically increased demand, but whether future demand will continue to rise, or level out remains to be seen. Reimbursement of costs by PhilHealth for reallocation by local governments is not yet having a significant impact and the higher cost of vaccine to purchasers other than the national DOH significantly increases overall costs. Patient out of pocket expenses are significantly impacted by having to purchase vaccine and RIG. Thus, although there might be equality in access to ABTCs, there was considerable inequality amongst communities in their access to PEP at the cheapest price. Overburdened ABTCs with large patient loads can suffer vaccine stock-outs and incur high OOPE for patients, which could affect their willingness to complete the PEP course. In the situation of the Philippines, private supplies of vaccine are relatively easy to access, but in other countries relying on government vaccine supply, this would be a significant concern.

There was evidence that several ABTCs lacked the full complement of recently trained staff, and data reporting discrepancies point to the need to strengthen reporting systems to ensure that more accurate data is available. All of the ABTCs were currently producing paper-based records which created a backlog of entries to the computer system in the highest throughput clinics. Stricter implementation of record submission and stricter implementation of the updated National Rabies Information System (NaRIS) should allow more accurate data on human rabies and bites to be both collected from and distributed back to all levels, increasing the value of the data and strengthening the control network considerably.

### Distributions of ABTCS and rabies deaths

The number and strategic placement of ABTCs is an important factor in PEP delivery. The data collected here provide some evidence that provinces with higher numbers of ABTCs per 100,000 population reduces human death incidence. Palawan, which has 21 ABTCs, has seen 0 human rabies since 2014. In theory, strategically placed ABTCs will reduce the costs of transport for patients and could improve health seeking behaviour.

However, despite a downward trend in human rabies case incidences as ABTC provision increased, there was no simple negative relationship across all provinces. This would also help to explain why significant increases in access to PEP through the expansion of the ABTC network over recent years has not had a similar significant impact on human deaths at the country level. The fact that many rabies deaths still occur close to the location of ABTCs suggests that other factors are at play.

The current analysis found that 27–8%about a third of human rabies deaths were under 15 years old, and that 72% were in males. These data are similar to those from patients at the Research Institute for Tropical Medicine, a government referral hospital for infectious diseases, where 27% of deaths occurred among patients below 15 years—compared to 40% in the past (Beatriz Quiambao, personal communication, 10 November 2017). This trend suggests that children are more often receiving life-saving PEP than in the past. The apparent trend of higher rabies incidences amongst higher income provinces is not fully explained, and the income level of the patient’s family may reveal different patterns to the general characteristics of the municipality analysed here. It appears not to be due to a lack of ABTC provision, as this is relatively constant across population densities and income classifications. It is possible that rabies deaths are more likely to be reported in wealthier provinces.

To provide an ABTC network that satisfies the goal of providing 1 ABTC per 100,000 population and which treats every person bitten by a rabies suspect animal would require considerable increases in budget. On top of existing costs, this would need to (a) pay for 100% of the currently demanded vaccine in existing ABTCs, (b) staff and supply a further 564 ABTCs to reach the goal of 1 ABTC / 100,000 population in all provinces, and (c) potentially cope with a higher proportion of bite victims presenting for treatment.

Since PEP provision is only one part of a comprehensive strategy for rabies control, it is likely that interactions with other factors (such as geography that may impact upon the baseline incidence of rabies in dogs, and dog vaccination rates) also have an impact on the incidence of human deaths. More detailed analysis of these aspects would be warranted to explore this further. Additionally, if rabies elimination is to be reached, it will be important to fully investigate the details of each human case, to ascertain the factors contributing to the death.

### More judicious use of PEP

Alongside the use of the intradermal delivery for PEP, reductions in the number of PEP doses used for previously vaccinated patients and those exposed to dogs that remained healthy, bears testimony to some judicious use of vaccines. Additionally, the Provincial Health Office in Nueva Vizcaya provides more PVRV (0.5 mL) vials than PCEC (1.0 mL) to their low volume ABTCS to reduce vaccine wastage. It was also reported from Nueva Vizcaya that not all Category 3 patients were prescribed RIG, with the severity and location of wound, and the number of days from bite to consultation being some of factors considered for deciding if a Category 3 patient was prescribed RIG or not.

However, in one ABTC, vaccine was being administered intramuscularly when the number of patients seen was fewer than the recommended number of patients sharing a vial under the intradermal route. This practice actually excludes any possibility of vial sharing (should another patient present for PEP the same day), and therefore will not reduce vaccine wastage at all.

Very recently, a proposed series of changes to the guidelines on the use of rabies vaccines and RIG were presented to the WHO Strategic Advisory Group of Experts (SAGE) committee [11]. These recommendations were approved, and include a shorter 3 visit (6 dose) schedule for regular PEP by the intradermal route, prioritization of cases for RIG administration, and injection of RIG into the wound only. The Philippines will have further opportunities to reduce PEP costs when these new guidelines are adopted.

Withholding or delaying PEP where a risk assessment of the biting animal can be carried out or where the animal can be held for observation is currently the subject of debate and some controversy in canine rabies endemic countries. Where canine rabies is well controlled by vaccination (e.g. USA [12] and Canada [13]) and in some states of Mexico, the guidance is that any animal that was healthy at the time of a bite should be confined for 10 days and PEP is not given unless signs of illness in the animal develop. In rabies endemic countries there is anecdotal evidence that similar informal risk assessments are carried out where extremely short supplies of vaccine necessitate it, but where vaccine supplies are adequate and rabies is highly endemic this is not practiced.

Less controversial is the withholding of PEP in instances where proof of vaccination of the biting animal is available. There is precedent for this in the national guidelines for the Philippines [14] which states that in the event of a bite from a well-documented vaccinated animal, PEP can be delayed pending observation. In practice however, adequate evidence of vaccination is rarely produced. This suggests that strengthening dog vaccination programs and ensuring adequate recording of vaccination would be necessary in order to utilise this mechanism to reduce PEP usage. Since dog observation is already being used to stop PEP after the third dose, some type of observation of the biting animal could perhaps be used to reduce PEP doses even further. In Haiti, an intensive integrated bite case management process is enabling the determination of the relative risk of individual exposures, paving the way for more targeted use of PEP regimes where the dogs can be safely observed [15]. In Sri Lanka where high coverage dog vaccination is established, PEP is now withheld pending observation of the dog [16].

### A more integrated rabies control strategy

Whilst critical and life-saving in the event of a rabies exposure, PEP provision remains just one piece of an integrated strategy for rabies control, which also involves community awareness, dog bite prevention and responsible dog ownership and mass dog vaccination. Extensive rabies awareness initiatives, including the integration of rabies information into the school curriculum may reduce bite incidences, encourage dog vaccination and improve treatment seeking behaviour to reduce the risk of rabies in communities and allow more judicious use of PEP.

Dividing resources optimally between these different strands will not only improve the cost effectiveness of the whole rabies prevention program, but is also the only way to reach the elimination of rabies from dogs and therefore an end to the threat of rabies to human health and the considerable financial burden that it incurs.

## Supporting information

S1 Fig Recent case incidence (2014–16) vs (A) ABTC density and (B) human population density for provinces. (TIF)

S2 Fig Mean ±SE of recent case incidence for municipalities by income class. (TIF)

S1 Table (A) Documents and datasets reviewed and (B) Key informant interviews conducted to understand the operation of ABTCs in the Philippines. (DOCX)

S2 Table ABTCs and rabies deaths per province, based on national data. (DOCX)

S3 Table Summary of ABTC operational costs and funders for study ABTCs in 2016. (DOCX)
