# Supplementary figures and images for: The evaluation of operating Animal Bite Treatment Centers in the Philippines from a health provider perspective
Source: PLoS One. 2018 Jul 12;13(7):e0199186. doi: 10.1371/journal.pone.0199186 (PMC6042697; doi:10.1371/journal.pone.0199186)

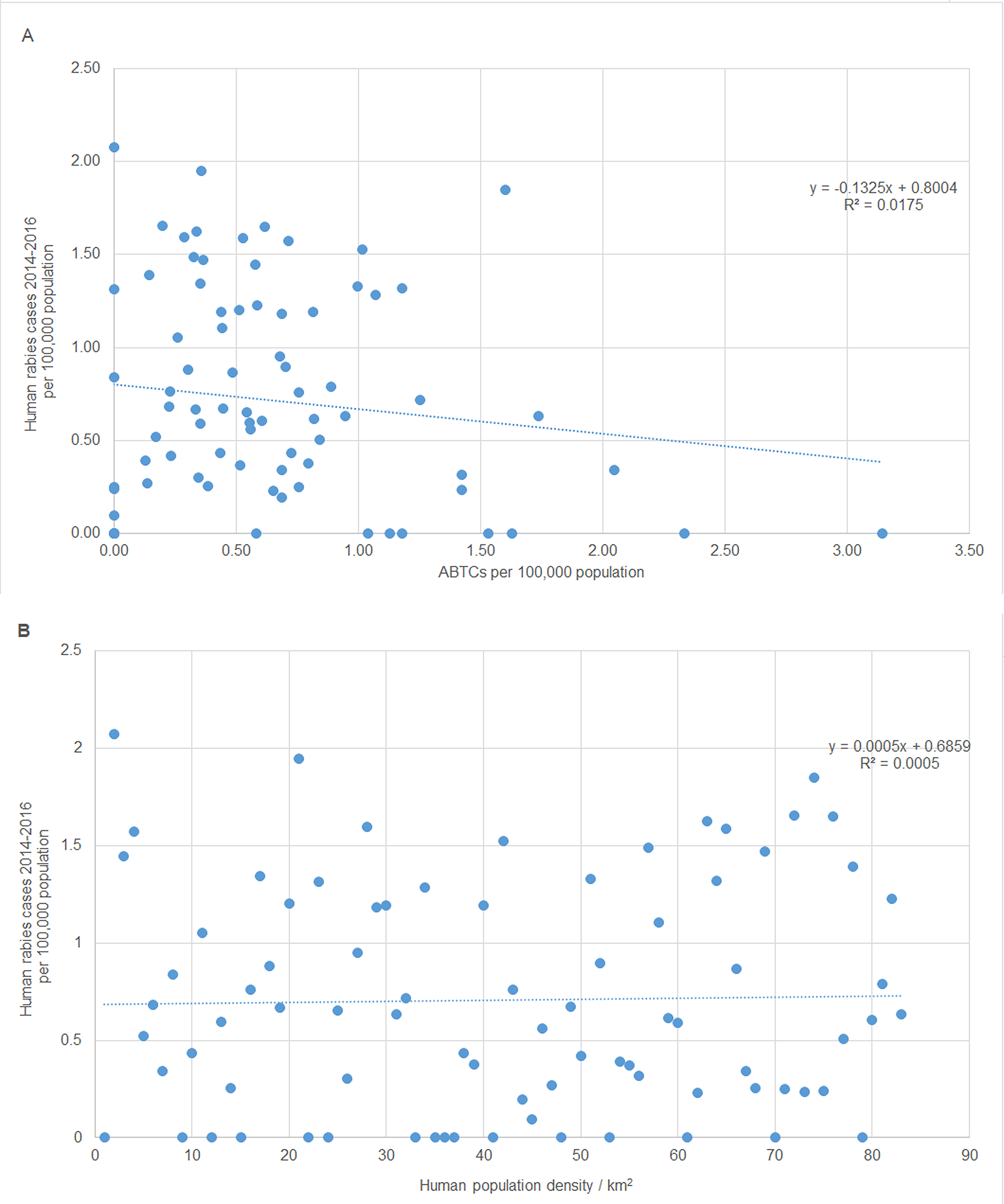

Supplement: S1 Fig — (TIF) [file pone.0199186.s001.tif]

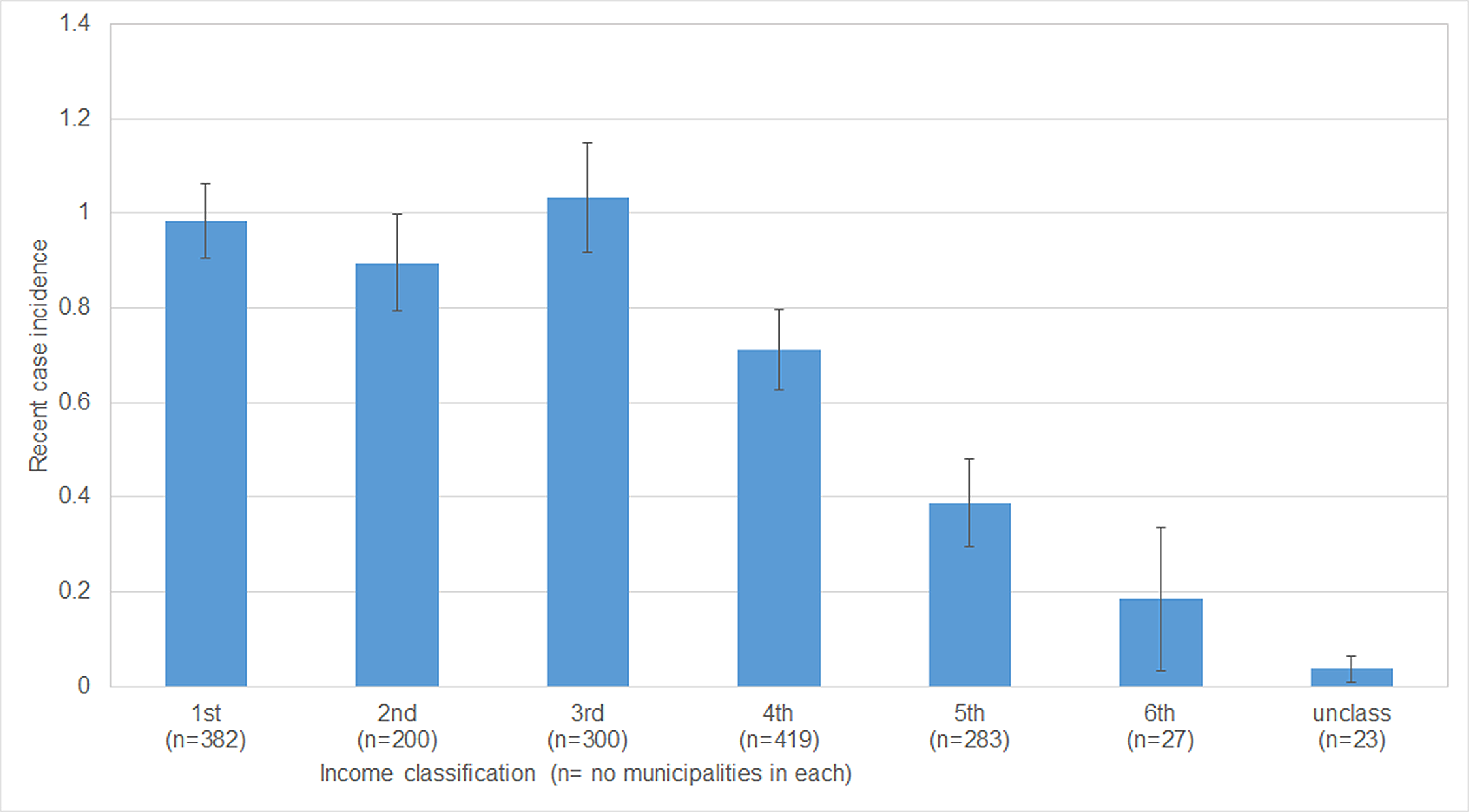

Supplement: S2 Fig — (TIF) [file pone.0199186.s002.tif]
