# Supplementary material for: The evaluation of operating Animal Bite Treatment Centers in the Philippines from a health provider perspective
Source: PLoS One. 2018 Jul 12;13(7):e0199186. doi: 10.1371/journal.pone.0199186 (PMC6042697; doi:10.1371/journal.pone.0199186)
Supplement: S1 Table — (DOCX) [file pone.0199186.s003.docx]

| 1. Document / Data set | Source |
| --- | --- |
| Anti-Rabies Act of 2007, Republic Act 9482 , An Act Providing for the Control and Elimination of Human and Animal Rabies, Prescribing Penalties for Violation Thereof and Appropriating Funds thereof | http://www.officialgazette.gov.ph/downloads/2007/05may/20070525-RA-9482-GMA.pdf |
| DOH Administrative Order No. 2013-004, Implementing Guidelines on the Conduct of Animal Bite Treatment Centers (ABTC) and Animal Bite Centers Certification of the National Rabies Prevention and Control Program | http://www.doh.gov.ph/sites/default/files/basic-page/ao2014-0012.pdf |
| DOH Administrative Order No. 2014-0012, New Guidelines on the Management of Rabies Exposures | http://www.doh.gov.ph/sites/default/files/basic-page/ao2014-0012.pdf |
| DOH-DA Administrative Order No. 2011-02, Guidelines for Managing Rabies Exposures Secondary to Bites by Vaccinated Dogs and Cats | http://rabies.chits.ph/index.php/13-documents/19-guidelines001 |
| DOH, DA, Dep Ed, DILG, National Rabies Prevention and Control Program Manual of Operations 2012 | http://www.doh.gov.ph/sites/default/files/publications/FINALMOP6.4.13WORDRADMay30.pdf |
| DOH, National Rabies Prevention and Control Program Medium-Term Plan 2012-2016 | http://www.doh.gov.ph/sites/default/files/publications/FINAL_MTP_Rabies.pdf |
| Joint DA, DOH, DILG Administrative Order No. 01, Series of 2008 Implementing Rules and Regulation Implementing Republic Act 9482, An Act Providing for the Control and Elimination of Human and Animal Rabies, Prescribing Penalties for Violation Thereof and Appropriating Funds thereof | http://www.lawphil.net/statutes/repacts/ra2007/ra_9482_2007.html |
| Philippine Health Insurance Corporation Circular 15 Series of 2012, PhilHealth for Animal Bite Package (Rabies Post Exposure Prophylaxis) | https://www.philhealth.gov.ph/circulars/2012/circ15_2012.pdf |
| Annual report of canine rabies from 2012-2016 | Bureau of Animal Industry, Department of Agriculture |
| List of ABTCs as of 6 July 2017  Historical ABTC numbers  Human animal bites treated from 2007-2015 from the DOH Annual Rabies and Bite Victim Report | Disease Prevention and Control Bureau (DPCB), DOH |
| Bite cases and human rabies cases for Nueva Vizcaya, Tarlac and Palawan  Vaccine volume, cold storage and distribution statistics | DOH Regional Offices II, III and IV-B |
| Human rabies data from 2008-2016 from Philippine Integrated Disease Surveillance and Response national database, Public Health Surveillance Division | Epidemiology Bureau, DOH |
| Annual report of ABTCs that availed of the PhilHealth animal bite package in 2016 | Philippine Health Insurance Corporation |
| Provincial dog vaccination records | Provincial Veterinary Offices of Nueva Vizcaya, Tarlac and Palawan |
| ABTC records on quarterly and annual bite victims reports, inventory of vaccines, staff and budgetary needs | Provincial Health Offices of Nueva Vizcaya, Tarlac and Palawan and 6 ABTC study sites |
| Vaccine volume, cold storage and distribution statistics | Research Institute for Tropical Medicine |

| **(B) Level** | **Office** | **Stakeholder interviewed** |
| --- | --- | --- |
| **National** | DOH National | National Rabies Program Coordinator and Team |
| **National** | Research Institute in Tropical Medicine | Chief of Clinical Research Division,  Pharmacist III, Cold Chain Personnel |
| **Regional** | DOH Region II | Regional Director, Regional Rabies Medical Coordinator, Cold Chain Personnel |
| **Regional** | DOH Region IVB | Regional Rabies Medical Coordinator, Regional Rabies Nurse Coordinator, Cold Chain Personnel |
| **Regional** | DOH Region III | Regional Rabies Coordinator, Cold Chain Personnel |
| **Provincial** | PHO Palawan | Provincial Rabies Coordinator, Provincial Health Officer, Cold Chain Personnel |
| **Provincial** | PHO Tarlac | Provincial Rabies Coordinator |
| **Provincial** | PHO Nueva Vizcaya | Provincial Rabies Coordinator, Administrative Assistant / Cold Chain Personnel |
| **ABTC** | Paniqui General Hospital | Chief of Hospital, ABTC Nurse |
| **ABTC** | Ospital ng Palawan | ABTC nurse, Cold Chain Personnel |
| **ABTC** | Alfonso Castañeda RHU | Municipal Health Officer, Municipal Rabies Coordinator, ABTC Midwife |
| **ABTC** | Southern Palawan Provincial Hospital | ABTC Nurse, Cold Chain Personnel |
| **Barangays** | Brgy. Bacungan, Puerto Princesa City, Palawan | Barangay Health Worker |
| **Barangays** | Brgy. San Miguel, PPC, Palawan | Barangay Secretary |
| **Barangays** | Brgy. Surgui 1st, Camiling Tarlac | Kagawad for Health |
| **Barangays** | Brgy. San Rafael, Tarlac City | Barangay Health Worker |
| **Barangays** | Pilar D. Galima, Solano, Nueva Vizcaya | Barangay Captain |
| **Barangays** | Osmeña, Solano, Nueva Vizcaya | Barangay Secretary |
