# Supplementary material for: The evaluation of operating Animal Bite Treatment Centers in the Philippines from a health provider perspective
Source: PLoS One. 2018 Jul 12;13(7):e0199186. doi: 10.1371/journal.pone.0199186 (PMC6042697; doi:10.1371/journal.pone.0199186)
Supplement: S2 Table — (DOCX) [file pone.0199186.s004.docx]

| **Province** | **Population (2015 census)** | **Income level** | **ABTC** | **ABTCs /100,000 popn.** | **Human Rabies cases 2008-13** | **Human Rabies Cases 2014-16** | **2014-16 case incidence / 100,000** | **Animal Rabies Cases**  **2014-16** |
| --- | --- | --- | --- | --- | --- | --- | --- | --- |
| Abra | 241,160 | 3 | 0 | 0.00 | 0 | 5 | 2.07 | 6 |
| Agusan del norte | 691,566 | 3 | 4 | 0.58 | 7 | 10 | 1.45 | 42 |
| Agusan del sur | 700,653 | 1 | 5 | 0.71 | 16 | 11 | 1.57 | 12 |
| Aklan | 574,823 | 2 | 1 | 0.17 | 4 | 3 | 0.52 | 4 |
| Albay | 1,314,826 | 1 | 3 | 0.23 | 43 | 9 | 0.68 | 14 |
| Antique | 582,012 | 2 | 4 | 0.69 | 5 | 2 | 0.34 | 14 |
| Apayao | 119,184 | 3 | 0 | 0.00 | 0 | 1 | 0.84 | 3 |
| Aurora | 214,336 | 3 | 5 | 2.33 | 0 | 0 | 0.00 | 0 |
| Basilan | 459,367 | 3 | 2 | 0.44 | 0 | 2 | 0.44 | 4 |
| Bataan | 760,650 | 1 | 2 | 0.26 | 1 | 8 | 1.05 | 48 |
| Batanes | 17,246 | 5 | 0 | 0.00 | 0 | 0 | 0.00 | 0 |
| Batangas | 2,694,335 | 1 | 15 | 0.56 | 38 | 16 | 0.59 | 25 |
| Benguet | 791,590 | 2 | 0 | 0.00 | 0 | 2 | 0.25 | 10 |
| Biliran | 171,612 | 4 | 1 | 0.58 | 4 | 0 | 0.00 | 2 |
| Bohol | 1,313,560 | 1 | 3 | 0.23 | 1 | 10 | 0.76 | 11 |
| Bukidnon | 1,415,226 | 1 | 5 | 0.35 | 50 | 19 | 1.34 | 14 |
| Bulacan | 3,292,071 | 1 | 10 | 0.30 | 86 | 29 | 0.88 | 94 |
| Cagayan | 1,199,320 | 1 | 4 | 0.33 | 24 | 8 | 0.67 | 20 |
| Camarines Norte | 583,313 | 2 | 3 | 0.51 | 41 | 7 | 1.20 | 13 |
| Camarines Sur | 1,952,544 | 1 | 7 | 0.36 | 67 | 38 | 1.95 | 26 |
| Camiguin | 88,478 | 5 | 1 | 1.13 | 0 | 0 | 0.00 | 0 |
| Capiz | 761,384 | 1 | 0 | 0.00 | 8 | 10 | 1.31 | 11 |
| Catanduanes | 260,964 | 3 | 4 | 1.53 | 9 | 0 | 0.00 | 1 |
| Cavite | 3,678,301 | 1 | 20 | 0.54 | 51 | 24 | 0.65 | 81 |
| Cebu | 4,632,359 | 1 | 16 | 0.35 | 26 | 14 | 0.30 | 137 |
| Compostela Valley | 736,107 | 1 | 5 | 0.68 | 20 | 7 | 0.95 | 7 |
| Cotabato | 1,379,747 | 1 | 4 | 0.29 | 50 | 22 | 1.59 | 1 |
| Davao del Norte | 1,016,332 | 1 | 7 | 0.69 | 25 | 12 | 1.18 | 6 |
| Davao del Sur | 2,265,579 | 1 | 10 | 0.44 | 39 | 27 | 1.19 | 48 |
| Davao Occidental | 316,342 | 4 | 3 | 0.95 | 2 | 2 | 0.63 | 0 |
| Davao Oriental | 558,958 | 1 | 7 | 1.25 | 4 | 4 | 0.72 | 0 |
| Dinagat Islands | 127,152 | 4 | 4 | 3.15 | 1 | 0 | 0.00 | 0 |
| Eastern Samar | 467,160 | 2 | 5 | 1.07 | 8 | 6 | 1.28 | 4 |
| Guimaras | 174,613 | 4 | 0 | 0.00 | 0 | 0 | 0.00 | 0 |
| Ifugao | 202,802 | 3 | 0 | 0.00 | 1 | 0 | 0.00 | 5 |
| Ilocos Norte | 593,081 | 1 | 7 | 1.18 | 12 | 0 | 0.00 | 0 |
| Ilocos Sur | 689,668 | 1 | 5 | 0.72 | 16 | 3 | 0.43 | 1 |
| Iloilo | 2,384,415 | 1 | 19 | 0.80 | 29 | 9 | 0.38 | 37 |
| Isabela | 1,593,566 | 1 | 13 | 0.82 | 52 | 19 | 1.19 | 17 |
| Kalinga | 212,680 | 3 | 0 | 0.00 | 1 | 0 | 0.00 | 13 |
| La Union | 786,653 | 1 | 8 | 1.02 | 13 | 12 | 1.53 | 24 |
| Laguna | 3,035,081 | 1 | 23 | 0.76 | 61 | 23 | 0.76 | 53 |
| Lanao del Norte | 1,019,013 | 2 | 7 | 0.69 | 6 | 2 | 0.20 | 8 |
| Lanao del Sur | 1,045,429 | 1 | 0 | 0.00 | 2 | 1 | 0.10 | 0 |
| Leyte | 1,966,768 | 1 | 11 | 0.56 | 18 | 11 | 0.56 | 9 |
| MAGUINDANAO | 1,473,371 | 1 | 2 | 0.14 | 5 | 4 | 0.27 | 4 |
| Marinduque | 234,521 | 4 | 0 | 0.00 | 1 | 0 | 0.00 | 0 |
| Masbate | 892,393 | 1 | 4 | 0.45 | 18 | 6 | 0.67 | 0 |
| Metro Manila | 12,877,253 | special | 30 | 0.23 | 108 | 54 | 0.42 | 254 |
| Misamis Occidental | 602,126 | 2 | 6 | 1.00 | 5 | 8 | 1.33 | 5 |
| Misamis Oriental | 1,564,459 | 1 | 11 | 0.70 | 10 | 14 | 0.89 | 116 |
| Mountain Province | 154,590 | 4 | 0 | 0.00 | 5 | 0 | 0.00 | 3 |
| Negros Occidental | 3,059,136 | 1 | 4 | 0.13 | 12 | 12 | 0.39 | 62 |
| Negros Oriental | 1,354,995 | 1 | 7 | 0.52 | 5 | 5 | 0.37 | 26 |
| Northern Samar | 632,379 | 2 | 9 | 1.42 | 11 | 2 | 0.32 | 1 |
| North Cotabato |  |  |  |  |  |  |  | 6 |
| Nueva Ecija | 2,151,461 | 1 | 7 | 0.33 | 34 | 32 | 1.49 | 49 |
| Nueva Vizcaya | 452,287 | 2 | 2 | 0.44 | 17 | 5 | 1.11 | 9 |
| Occidental Mindoro | 487,414 | 2 | 4 | 0.82 | 9 | 3 | 0.62 | 2 |
| Oriental Mindoro | 844,059 | 1 | 3 | 0.36 | 20 | 5 | 0.59 | 7 |
| Palawan | 1,104,585 | 1 | 18 | 1.63 | 20 | 0 | 0.00 | 17 |
| Pampanga | 2,609,744 | 1 | 17 | 0.65 | 10 | 6 | 0.23 | 221 |
| Pangasinan | 2,956,726 | 1 | 10 | 0.34 | 51 | 48 | 1.62 | 186 |
| Quezon | 2,122,830 | 1 | 25 | 1.18 | 46 | 28 | 1.32 | 29 |
| Quirino | 188,991 | 3 | 1 | 0.53 | 6 | 3 | 1.59 | 1 |
| Rizal | 2,884,227 | 1 | 14 | 0.49 | 46 | 25 | 0.87 | 79 |
| Romblon | 292,781 | 3 | 6 | 2.05 | 11 | 1 | 0.34 | 0 |
| Samar | 780,481 | 1 | 3 | 0.38 | 19 | 2 | 0.26 | 2 |
| Sarangani | 544,261 | 2 | 2 | 0.37 | 12 | 8 | 1.47 | 12 |
| Siquijor | 95,984 | 5 | 1 | 1.04 | 0 | 0 | 0.00 | 0 |
| Sorsogon | 792,949 | 2 | 6 | 0.76 | 13 | 2 | 0.25 | 6 |
| South Cotabato | 1,509,735 | 1 | 3 | 0.20 | 25 | 25 | 1.66 | 78 |
| Southern Leyte | 421,750 | 3 | 6 | 1.42 | 1 | 1 | 0.24 | 6 |
| Sultan Kudarat | 812,095 | 1 | 13 | 1.60 | 21 | 15 | 1.85 | 12 |
| Sulu | 824,731 | 2 | 0 | 0.00 | 2 | 2 | 0.24 | 0 |
| Surigao del Norte | 485,088 | 2 | 3 | 0.62 | 7 | 8 | 1.65 | 10 |
| Surigao del Sur | 592,250 | 1 | 5 | 0.84 | 6 | 3 | 0.51 | 11 |
| Tarlac | 1,366,027 | 1 | 2 | 0.15 | 28 | 19 | 1.39 | 12 |
| Tawi-Tawi | 390,715 | 3 | 0 | 0.00 | 0 | 0 | 0.00 | 0 |
| Zambales | 823,888 | 2 | 5 | 0.61 | 3 | 5 | 0.61 | 20 |
| Zamboanga del Norte | 1,011,393 | 1 | 9 | 0.89 | 11 | 8 | 0.79 | 1 |
| Zamboanga del Sur | 1,872,473 | 1 | 11 | 0.59 | 15 | 23 | 1.23 | 63 |
| Zamboanga Sibugay | 633,129 | 2 | 11 | 1.74 | 11 | 4 | 0.63 | 1 |
