# Supplementary material for: The evaluation of operating Animal Bite Treatment Centers in the Philippines from a health provider perspective
Source: PLoS One. 2018 Jul 12;13(7):e0199186. doi: 10.1371/journal.pone.0199186 (PMC6042697; doi:10.1371/journal.pone.0199186)
Supplement: S3 Table — (DOCX) [file pone.0199186.s005.docx]

**ABTC Operational Expenses by type of input and funding source, Nueva Vizcaya, 2016**

| Type of Input | Urban ABTC | | | | | Rural ABTC | | | | |
| --- | --- | --- | --- | --- | --- | --- | --- | --- | --- | --- |
|  | DOH | LGU | OOPE | Others | Total | DOH | LGU | OOPE | Others | Total |
| Personnel | 1,215 | 17,717 | 0 | 2,139 | 21,072 | 1,215 | 4,934 | 0 | 0 | 6,149 |
| Training of ABTC staff in Bite Mgt | 501 | 399 | 0 | 0 | 900 | 115 | 106 | 0 | 0 | 221 |
| Rabies Vaccines (TCV & RIG) | 51,503 | 22,207 | 35,857 | 0 | 109,567 | 2,378 | 1,256 | 1,290 | 0 | 4,925 |
| Other consumables | 299 | 492 | 3,902 | 0 | 4,692 | 10 | 0 | 184 | 0 | 195 |
| Vaccine Distribution Costs | 4,522 | 0 | 0 | 0 | 4,522 | 240 | 407 | 0 | 0 | 647 |
| Vaccine Storage costs | 1,184 | 210 | 0 | 0 | 1,394 | 59 | 7 | 0 | 0 | 67 |
| ABTC Equipment | 2,948 | 0 | 0 | 2,034 | 4,982 | 89 | 1 | 0 | 96 | 186 |
| Information, Education, Communications | 5 | 0 | 0 | 0 | 5 | 0 | 21 | 0 | 0 | 21 |
| Total (USD) | 62,177 | 41,026  (28%) | 39,759  (27%) | 4,173 | 147,135 | 4,106 | 6,732  (54%) | 1,474  (12%) | 96 | 12,411 |

**ABTC Operational Expenses by type of input and funding source, Palawan, 2016** (The Urban ABTC in Palawan is a DOH-retained hospital, and all staff costs are shouldered by DOH.

| Type of Input | Urban ABTC | | | | | Rural ABTC | | | | |
| --- | --- | --- | --- | --- | --- | --- | --- | --- | --- | --- |
|  | DOH | LGU | OOPE | Others | Total | DOH | LGU | OOPE | Others | Total |
| Personnel | 8,684 | 0 | 0 | 0 | 8,684 | 124 | 8,924 | 0 | 0 | 9048 |
| Training of ABTC staff in Bite Mgt | 1,159 | 0 | 0 | 0 | 1,159 | 255 | 490 | 0 | 0 | 745 |
| Rabies Vaccines (TCV & RIG) | 14,474 | 0 | 21,846 | 0 | 36,320 | 3,916 | 161 | 13,723 | 0 | 17801 |
| Other consumables | 150 | 0 | 3,824 | 0 | 3,974 | 41 | 0 | 4,481 | 127 | 4649 |
| Vaccine Distribution Costs | 1,635 | 0 | 0 | 0 | 1,635 | 321 | 13 | 0 | 0 | 334 |
| Vaccine Storage costs | 134 | 0 | 0 | 0 | 134 | 11 | 43 | 0 | 0 | 54 |
| ABTC Equipment | 741 | 106 | 0 | 7 | 854 | 116 | 90 | 0 | 4,913 | 5120 |
| Information, Education, Communications | 4,244 | 0 | 0 | 5 | 4,250 | 0 | 85 | 0 | 0 | 85 |
| Total (USD) | 31,221 | 106  (0%) | 25,670  (45%) | 13 | 57,010 | 4,784 | 9,806  (26%) | 18,205  (46%) | 5,040 | 37,836 |

**ABTC Operational Expenses by type of input and funding source, Tarlac, 2016**

| Type of Input | **Urban ABTC** | | | | | **Rural ABTC** | | | | |
| --- | --- | --- | --- | --- | --- | --- | --- | --- | --- | --- |
|  | **DOH** | **LGU** | **OOPE** | **Others** | **Total** | **DOH** | **LGU** | **OOPE** | **Others** | **Total** |
| **Personnel** | 978 | 35,856 | 0 | 0 | 36,834 | 978 | 6,347 | 0 | 0 | 7,325 |
| **Training of ABTC staff in Bite Mgt** | 115 | 0 | 0 | 0 | 115 | 272 | 0 | 0 | 0 | 272 |
| **Rabies Vaccines (TCV & RIG)** | 35,945 | 95,115 | 152,466 | 0 | 283,525 | 889 | 2,037 | 764 | 0 | 3,691 |
| **Other consumables** | 448 | 101 | 9,060 | 0 | 9,609 | 9 | 0 | 511 | 0 | 520 |
| **Vaccine Distribution Costs** | 3,542 | 98 | 0 | 0 | 3,640 | 86 | 70 | 0 | 0 | 156 |
| **Vaccine Storage costs** | 482 | 1,167 | 0 | 0 | 1,649 | 1,530 | 2,626 | 0 | 0 | 4,156 |
| **ABTC Equipment** | 14,358 | 552 | 0 | 1,253 | 16,163 | 1,011 | 5,336 | 0 | 1,995 | 8,342 |
| **Information, Education, Communications** | 1,545 | 0 | 0 | 0 | 1,545 | 0 | 8 | 0 | 0 | 8 |
| **Total (USD)** | 57,413 | 132,888  (38%) | 161,526  (46%) | 1,253 | 353,080 | 4,776 | 16,424  (67%) | 1,275  (5%) | 1,995 | 24,470 |
